# Molecular insights and rational engineering of a compact CRISPR-Cas effector Cas12h1 with a broad-spectrum PAM

**DOI:** 10.1038/s41392-025-02147-5

**Published:** 2025-02-12

**Authors:** Weiwei Zheng, Hongyu Li, Mengxi Liu, Yuhang Wei, Bo Liu, Zekai Li, Chenyang Xiong, Shiqing Huang, Chunyi Hu, Songying Ouyang

**Affiliations:** 1 Key Laboratory of Microbial Pathogenesis and Interventions of Fujian Province University, the Key Laboratory of Innate Immune Biology of Fujian Province, Biomedical Research Center of South China, Key Laboratory of OptoElectronic Science and Technology for Medicine of the Ministry of Education, College of Life Sciences, Fujian Normal University, Fuzhou, 350117 China; 2 Department of Biological Sciences, Faculty of Science; Department of Biochemistry, Precision Medicine Translational Research Programme (TRP), Yong Loo Lin School of Medicine, National University of Singapore, Singapore, 117557 Singapore

**Keywords:** Structural biology, Microbiology

## Abstract

Cas12h1 is a compact CRISPR-associated nuclease from functionally diverse type V CRISPR-Cas effectors and recognizes a purine-rich protospacer adjacent motif (PAM) distinct from that of other type V Cas effectors. Here, we report the nickase preference of Cas12h1, which predominantly cleaves the nontarget strand (NTS) of a double-stranded DNA (dsDNA) substrate. In addition, Cas12h1 acts as a nickase in human cells. We further determined the cryo-EM structures of Cas12h1 in the surveillance, R-loop formation, and interference states, revealing the molecular mechanisms involved in the crRNA maturation, target recognition, R-loop formation, nuclease activation and target degradation. Cas12h1 notably recognizes a broad 5’-DHR-3’ PAM (D is A, G, or T; H is A, C, or T; R is A or G) both in vitro and in human cells. In addition, Cas12h1 utilizes a distinct activation mechanism that the lid motif undergoes a “flexible to stable” transition to expose the catalytic site to the substrate. A high-fidelity nucleic acid detector, Cas12h1^hf^, was developed through rational engineering, which distinguishes single-base mismatches and retains comparable on-target activities. Our results shed light on the molecular mechanisms underlying Cas12h1 nickase, improve the understanding of type V Cas effectors, and expand the CRISPR toolbox for genome editing and molecular diagnosis.

## Introduction

Clustered Regularly Interspaced Short Palindromic Repeats (CRISPR) and CRISPR-associated (Cas) proteins constitute prokaryotic adaptive immune systems that defend against phages and other mobile genetic elements (MGEs) through RNA-guided cleavage of invasive nucleic acids.^1^ Some Cas effectors require additional RNAs (such as tracrRNA or scoutRNA) for crRNA maturation and target cleavage.^2^ The CRISPR-Cas system has been developed into groundbreaking genome editing technologies. It functions by generating double-strand DNA (dsDNA) breaks at desired sites, which are then repaired by error-prone non-homologous end joining (NHEJ) or error-free homology-directed repair (HDR) in eukaryotes. In addition to conventional genome editing, the nuclease-deficient Cas fused with other effectors or domains has expanded its applications to base editing, prime editing, and transcription regulation.^3,4^ The CRISPR-Cas have been harnessed in various areas encompassing clinical therapy, pharmaceutical production, molecular diagnosis crop improvement, and animal modeling.^4^

CRISPR-Cas systems are categorized into two classes—Class 1 and Class 2—based on the number of effector molecules.^1^ The class 1 CRISPR-Cas systems consist of multiple Cas proteins forming a complex, whereas the class 2 systems are featured by a single multi-domain protein with multiple functions, making them highly attractive for diverse genome editing applications. These classes are further divided into six types (I to VI), with the type II (Cas9), type V (Cas12), and type VI (Cas13) belonging to the class 2. Among them, the Cas9 from the type II system stands out for its widespread application, attributed to its precision, efficiency, and adaptability for engineering purposes.^5^ Despite its advantages, the utilization of Cas9 faces limitations, including its substantial size, which poses challenges for cellular delivery,^6^ the requirement of a specific Protospacer Adjacent Motif (PAM), which restricts target gene selection, and the limited fidelity due to its tolerance to the sequence with imperfect complementarity to the gRNA.^7^ Consequently, the exploration and characterization of new CRISPR-Cas systems is crucial for advancing therapeutic applications.

The type V CRISPR-Cas system, the Cas12 family, encompassing tens of subtypes from V-A to V-O, represents the most diverse family within the CRISPR-Cas repertoire.^8–10^ Varying in protein size from approximately 400 amino acids to 1400 amino acids, the type V Cas effectors are guided by single or dual RNAs to recognize dsDNA, single strand DNA (ssDNA) or single strand RNA (ssRNA). Upon activation by the target, most of these effectors degrade the on-target dsDNA (*cis*-cleavage), generating staggered ends, which is considered holding advantages for application such as integrating DNA sequences in a precise orientation.^4^ Meanwhile, active type V Cas effectors indiscriminately cleave ssDNA or ssRNA in the environment (*trans*-cleavage or collateral cleavage), based on which they have been developed into nucleic acid detectors.^11^ Given this diversity, a member of the type V system holds significant promise for unveiling novel functionalities and diversities. Within type V CRISPR systems, Cas12h1 stands out due to its unique characteristics. Firstly, it exhibits a notable variation in its PAM requirements. Unlike most Cas12 subtypes that prefer a 5ʹ T-rich PAM, Cas12h1 utilizes a purine-rich PAM sequence, 5’-RTR-3’ (where R represents A or G).^8^ This adaptation expands the repertoire of genomic sites amenable to editing. Secondly, as a member of subtype V-H, Cas12h1 is distinguished by its relatively small size (870 amino acids), functioning as a compact DNA endonuclease guided by a single crRNA with proficient DNA cleavage capability, highlighting its potential as an efficient genome editing tool. Lastly, the low sequence similarity Cas12h shares with other Cas12 effectors hints at a rich vein of functional diversity yet to be explored, possibly offering novel genome engineering opportunities. However, the characterization of the nuclease activity of Cas12h1 is unclear, except that Cas12h1 may asymmetrically cleave dsDNA according to the characteristics of its distant homologs, Cas12b^12^ and Cas12i.^8^ In addition, the underlying molecular mechanisms of Cas12h1 have not been elucidated since no structural information is available.

Here, we showed that Cas12h1 exhibits robust editing efficiency in human cells. Cas12h1 preferentially nicks the nontarget strand (NTS) of target dsDNA and indiscriminately cleaves ssDNA by collateral cleavage in the RuvC domain. Cryo-EM studies of the Cas12h1^WT^-crRNA surveillance complex, the Cas12h1^WT^-crRNA-dsDNA interference complex, and the Cas12h1^D465A^-crRNA-dsDNA R-loop formation complex depict the architecture of the Cas12h1 protein, and the molecular mechanisms of the crRNA maturation, PAM recognition, R-loop formation, and target degradation. Remarkably, we determined that Cas12h1 recognizes a 5ʹ-DHR-3ʹ (D is A, T, or G, and H is A, T, or C) PAM, which is a broader spectrum than the previously reported 5ʹ-RTR-3ʹ. The structural analysis also reveals a distinct “flexible-to-stable” transition of the lid motif in Cas12h1 for its activation. Furthermore, Cas12h1 is developed into a time-saving, equipment-free nucleic acid diagnostic tool and further improved to be a highly specific detector through rationally engineering. Overall, the findings of this study help characterize the functions of Cas12h1, provide insights into its molecular mechanisms, and reveal its promising potential applications as a genome editing tool as well as nucleic acid detector.

## Results

### Cas12h1 exhibits robust editing efficacy and demonstrates asymmetric cleavage in vivo

**Fig. 1:**
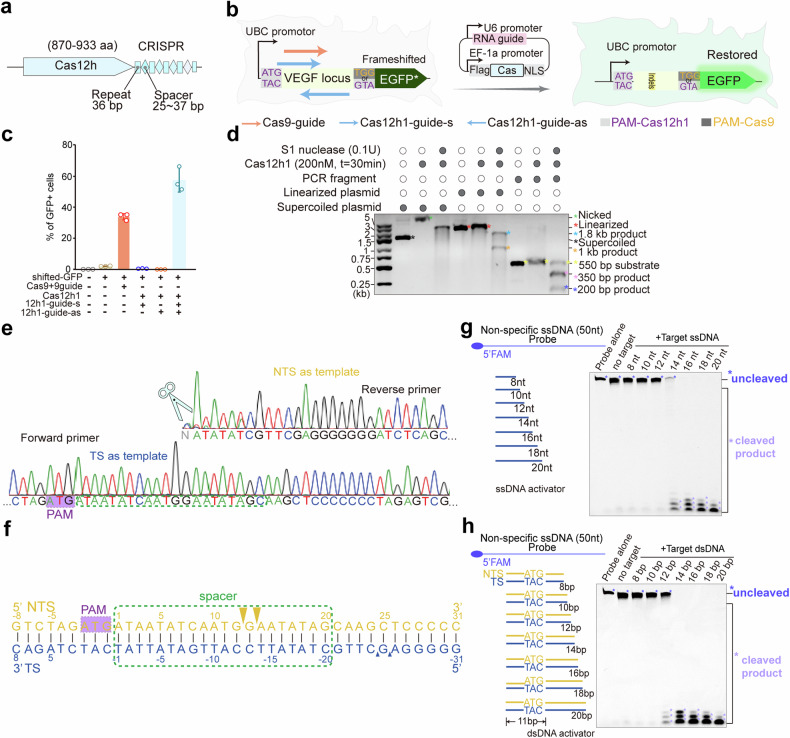
Cas12h1 exhibits robust editing efficacy and demonstrates asymmetric cleavage in human cells and in vitro. **a** Scheme illustrating the genomic locus of CRISPR–Cas12h1. aa amino acid. **b** Scheme of Cas12h1-mediated EGFP activation in human cells. **c** EGFP activation efficiencies by Cas12h1 with different guides determined by flow cytometry. Data represents mean ± SEM. of three biological replicates. Cas9 with its corresponding guides was used as a positive control. **d** S1 nuclease treatment to resolve dsDNA nicks induced by Cas12h1 into dsDNA breaks in supercoiled plasmids, linearized plasmids, or 550 bp PCR fragment. Linearized plasmids, the Cas12h1 supercoiled substrate digested by *Sca*I, which is 1 kb away from Cas12h1 recognition site. PCR fragment, a 550 bp fragment amplified from the Cas12h1 target plasmid. **e** Sanger sequencing results of supercoiled plasmids cleaved by Cas12h1 at a concentration of 200 nM for 30 min. The additional non-templated nucleotides are denoted as N in gray. The cleavage site is indicated by a scissor. **f** Scheme illustrating the cleavage pattern on a 40 bp dsDNA fragment by Cas12h1. The PAM and spacer region are highlighted in purple and green dashed lines, respectively. The cleavage sites identified under denaturing conditions are indicated by yellow (NTS) or blue (TS) triangles. Larger triangles indicated Cas12h1 exhibited a higher cleavage activity on the NTS. Evaluation of *trans*-cleavage triggered by ssDNA (**g**) or dsDNA (**h**) activators. Left: Schematic representations of ssDNA activators (**g**) and dsDNA activators (**h**) with different lengths. Right: the cleavage of the fluorescently labeled nonspecific ssDNA by Cas12h1 in the presence of different ssDNA (**g**) and dsDNA (**h**) activators

Cas12h1 was discovered from metagenomic data and exhibited plasmid interference activity in bacteria (Fig. 1a). To investigate the potential of Cas12h1 for genome editing, we performed a GFP activation assay by introducing two plasmids into HEK293T.^13^ A plasmid containing inactivate GFP cassette inserted by a short VEGF fragment sequence at the beginning of GFP coding sequence, leading to frameshift mutations. The indels generated by the Cas nucleases mediate frameshift mutations, which restore the previously frameshifted GFP cassette and leads to GFP expression (Fig. 1b). However, no fluorescence was observed when we co-transfected the frameshifted GFP with a plasmid encoding Cas12h1 and an RNA guide targeting one DNA strand (Cas12h1+guide-s, Fig. 1c). In consideration of the possibility that Cas12h1 asymmetrically cleaves the target DNA like its homologs, we further introduced another RNA guide targeting the antisense strand (Cas12h1+guide-as, Fig. 1b, c). When co-transfected with both Cas12h1+guide-s and Cas12h1+guide-as plasmids, up to 62% of cells exhibited editing and displayed a restored GFP signal (Fig. 1c). In addition, Cas12h1 exhibited higher editing efficiency than Cas12j2, another type V Cas effector with similar size (Supplementary Fig. 1a). The results suggest that Cas12h1 holds significant potential for development of a gene editor as a nickase.

### Cas12h1 exhibits asymmetric dsDNA cleavage in vitro

We further reconstituted the wild-type Cas12h1-crRNA binary complex (hereafter termed the surveillance complex) to identify the activity of Cas12h1. An in vitro cleavage assay showed that the purified Cas12h1-crRNA complex was able to cleave supercoiled plasmid DNA bearing a sequence complementary to the crRNA and adjacent to a 5’-ATG-3’ PAM at a minimal concentration of 50 nM and was active at a wide range of temperatures (Supplementary Figs. 1b, c). Comparison of the cleavage product of supercoil plasmid by Cas12h1 and linearized plasmid showed that it exhibited even lower electrophoretic mobility, indicating that Cas12h1 may generate nicks on supercoiled plasmids (Supplementary Fig. 1d). Further introduction of S1 nuclease, a single stranded DNA/RNA endonuclease, converts Cas12h1-treated supercoiled plasmids into linearized plasmids (Fig. 1d, Supplementary Fig. 1e). Accordingly, no evident cleavage product was found when a 2.8 kb linearized plasmid or a 550 bp PCR fragment was treated with Cas12h1 until the introduction of the S1 nuclease (Fig. 1d). In addition, Sanger sequencing of the cleaving product of the supercoiled plasmids revealed that Cas12h1 cuts at 13 nt downstream of the PAM sequence on the NTS DNA, but no cutting site on the target strand (TS) DNA was found (Fig. 1e). These results support the observations in mammalian cells that Cas12h1 preferably acts as a nickase.

To explore whether Cas12h1 cleaves the TS chain of a dsDNA, we increased the protein concentration as well as incubation time, and little dsDNA cleavage product was observed when the substrate was cleaved at a high protein concentration for overnight (Supplementary Fig. 1f), indicating that although weak, Cas12h1 retains the TS DNA cleavage ability in certain conditions, and the cleavage kinetics is slow. Taken together, Cas12h1 tends to act as a nickase in vitro except in extreme cases.

We further investigated the cleavage pattern of Cas12h1. With a high protein concentration at 4 μM, Cas12h1 cleaves the NTS DNA within 30 seconds, while minimal cleavage of the TS DNA is observed within 2 minutes, suggesting that Cas12h1 preferentially cleaves the NTS DNA (Supplementary Fig. 1g). Specifically, Cas12h1 cuts at 12 and 13 nucleotides downstream of the PAM duplex on the NTS DNA and at 24 and 25 nucleotides downstream of the PAM on TS DNA, generating a staggered cut (Fig. 1f, Supplementary Fig. 1g). This cleavage pattern is consistent with that of other type V CRISPR-Cas enzymes.^14,15^ To sum up, high concentration of Cas12h1 sequentially and asymmetrically cleaves dsDNA in vitro.

The *trans*-cleavage activity of Cas12h1 is further investigated by investigating whether a 50 nt FAM-labeled nonspecific ssDNA was cleaved with treatments of the dsDNA activators or the TS ssDNA activators. The results showed that a minimum length of 12 bp for the dsDNA or 14 nt for the ssDNA activator triggered the *trans*-cleavage activity of Cas12h1 (Fig. 1g, h). Furthermore, we performed a fluorophore-quencher (F-Q) assay to compare the activating efficiencies among different activators for Cas12h1. Among all the activators tested, dsDNA activators exhibited the highest activating efficiency (Supplementary Figs. 2a, b). In addition, we tested the optimum conditions for fluorescence detection. Our findings indicate that a lowest concentration of 5 nM Cas12h1-crRNA complex or 4 nM dsDNA activator produces a detectable signal. Furthermore, results suggest that a higher concentration of the FQ probe enhances detection (Supplementary Figs. 2c–e). Moreover, the concentration of activators determines cleavage kinetics, while the concentration of reporters determines maximum fluorescence (Supplementary Fig. 2f).

### Overall characterization of the Cas12h1^WT^-crRNA surveillance complex

To gain insight into the function of Cas12h1, we determined the structure of the surveillance complex using cryo-EM to a global resolution of 3.00 Å (Supplementary Fig. 3). Most residues of the PI domain (residues 103-173) are invisible in the surveillance complex, indicating its structural flexibility.

**Fig. 2:**
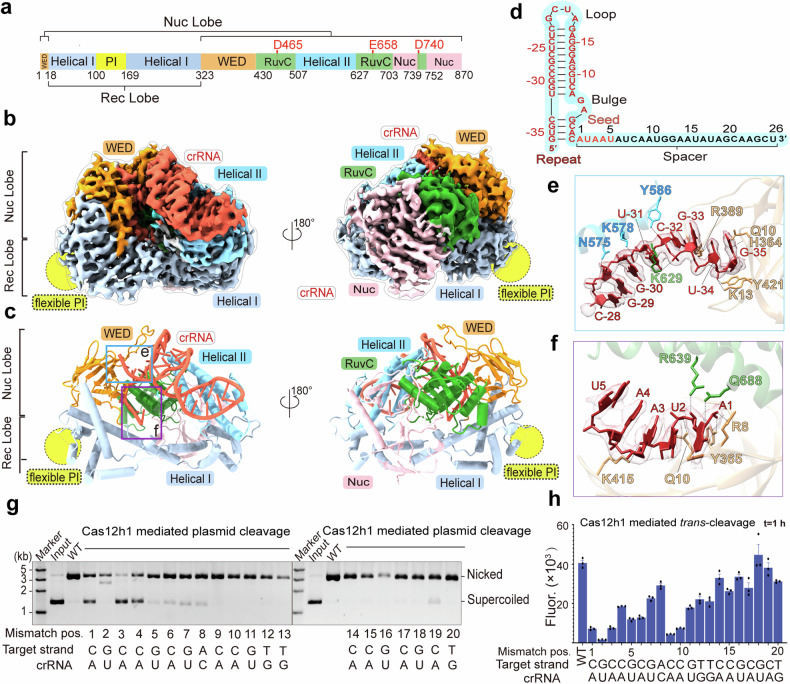
The Cas12h1-crRNA binary complex reveals pre-crRNA processing sites and pre-ordered seed region. **a** Domain organization of Cas12h1. **b** Cryo-EM map of the Cas12h1^WT^-crRNA surveillance complex in one view (left) and the 180° rotated view (right). Domains of Cas12h1 are colored as in (**a**), and the crRNA is colored in firebrick. **c** Atomic models of the Cas12h1^WT^-crRNA surveillance complex in two views in cartoon. **d** The sequence, and the secondary structure of the crRNA. Disordered regions are shown in gray color. The seed region is highlighted in a purple dashed rectangle. **e** Detailed interactions between the nucleotides G(-35)–G(-29) of the crRNA repeat region and Cas12h, with the density of nucleotides shown in mesh. Black dashed lines indicate hydrogen bonds. **f** The 5 nt pre-ordered crRNA seed region in Cas12h1^WT^-crRNA surveillance complex, with detailed interactions between crRNA and Cas12h1. Hydrogen bonds between Cas12h1 and crRNA are shown as black dashed lines. The density of nucleotides is shown in mesh. **g** Supercoiled plasmid cleavage testing the *cis*-cleavage activity of Cas12h1 with single mutated target DNA. **h** Mismatch F-Q assay detecting the *trans*-cleavage activity of Cas12h1 with single mutated target dsDNA activators (40 bp) with a 5’-ATG-3’ PAM

Cas12h1 adopts a bilobed architecture similar to that of other type V Cas effectors.^16–18^ Displaying a “Crab Claw” shape, Cas12h1 contains a distinguishable recognition (REC) lobe and a nuclease (NUC) lobe, with the two lobes connected by the WED domain (Fig. 2a–c). The REC lobe contains the dumbbell-like Helical I domain, which comprises a bundle of 9 α-helices and a flexible, putative PAM-interacting (PI) domain. The NUC lobe includes the WED, Helical II, RuvC, and Nuc domains. The WED domain is characterized by its ten-strand β-barrel, and the Helical II domain is made of 4 α-helix bundles. The RuvC domain folds into a central 5-strand twisted β-sheet flanked by 4 α-helices, with the catalytic sites D465, E658, and D740. The Nuc domain is positioned side-by-side with the RuvC domain and includes 2 α-helices and 5 β-sheets (Supplementary Fig. 4a).

A mature crRNA originating from a co-expressed CRISPR array template was determined in the complex. The mature crRNA forms an elongated stem-loop structure containing a 36 nt repeat-derived sequence larger than other type V crRNAs (positions –35 to 0; Fig. 2d).^15,18–20^ Another difference is there is no 5’ flank in the stem-loop of Cas12h1 crRNA. The stem-loop structure is held upon the surface of the Helical II domain, with its 5’-end inserting into a positively charged groove formed by the WED domain (Supplementary Figs. 4b, c), and is stabilized through numerous hydrogen bonds with Cas12h1 (Fig. 2e, Supplementary Figs. 4d, e and 10). The first nucleotide of the crRNA, G(-35), is recognized by the side chain of Q10 and the main chain of H364 (Fig. 2e). A 26 nt spacer-derived sequence follows the repeat region. The density of nucleotides 6 to 26 in the spacer region of the crRNA was not observed, indicating its flexibility in the surveillance state.

### A pre-ordered crRNA seed region for target recognition

The pre-ordered crRNA region is critical for target recognition by Cas effectors.^15,18^ The surveillance structure revealed that the first 1-5 nucleotides of the crRNA were the pre-ordered seed sequences. A detailed analysis of the protein-crRNA interaction showed that nucleotide A(1) forms hydrogen bonds with the side chains of R8, Q688, R639 (Fig. 2f). Cas effector-initiating target recognition relies on the correct match within the seed region^15^; therefore, we performed a single-nucleotide mismatch assay to investigate whether the seed region is critical for target recognition in Cas12h1. Single-nucleotide mismatches at positions 1, 3, and 4 significantly reduced both *cis*- and *trans*-cleavage activity. However, the mismatch at position 2 almost completely abolished the *trans*-cleavage activity but had little impact on the *cis*-cleavage activity (Fig. 2g, h). Accordingly, using another guide RNA targeting a bona fide target (the VP1 gene of the Norovirus) for mismatch paring, it comes to similar conclusions that mismatch at position 2 retains *cis*-cleavage activity but significantly reduce *trans*-cleavage activity (Supplementary Fig. 4f, g). This result indicates that the mismatch surveillance mechanism may differ between the two cleavages in Cas12h1. Possibly incorrect paring of crRNA and the TS triggers conformational change of Cas12h1 to an intermediate state, where the NTS is cut and trapped in the catalytic pocket that prevents nonspecific ssDNA entering for *trans*-cleavage.

### The architecture of the Cas12h1^WT^-crRNA-dsDNA interference complex reveals structural similarities among type V Cas effectors

**Fig. 3:**
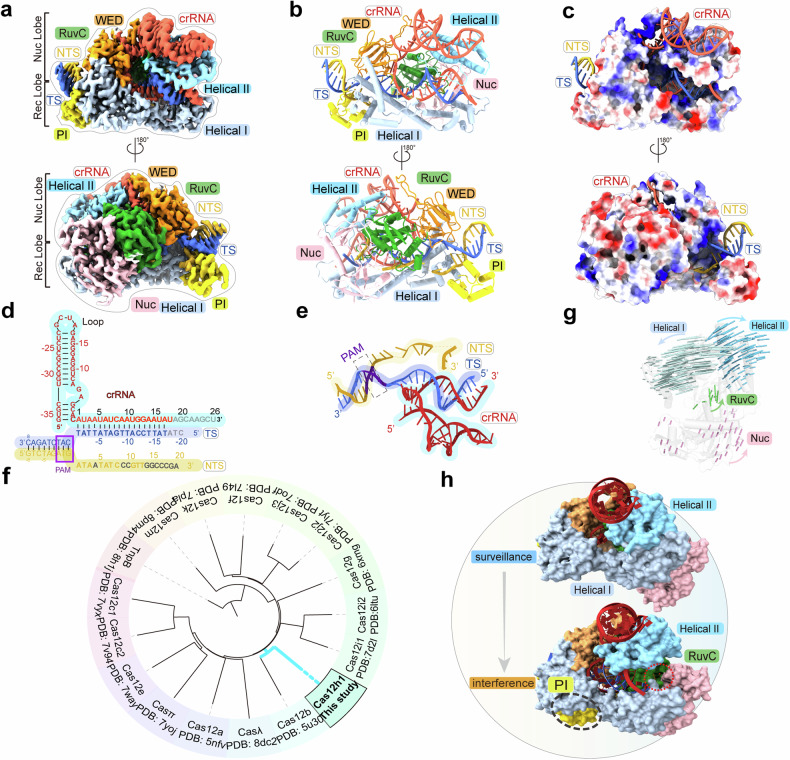
Target binding leads to conformational arrangement of Cas12h1. **a** Cryo-EM map of the Cas12h1^WT^-crRNA-dsDNA interference complex in one view (up) and the 180° rotated view (down). Domains of Cas12h1 are colored as in Fig. 2a, and the crRNA, TS, and NTS are colored in firebrick, royal blue, and dark goldenrod. **b** Atomic models of the Cas12h1^WT^-crRNA-dsDNA interference complex in two views in cartoon. **c** Electrostatic potential surface of Cas12h1 with crRNA and DNA. Red, white, and blue indicate surfaces with negative, neutral, and positive electrostatic potential, respectively. **d** Schematic representation of the R-loop structure formed by crRNA and target DNAs. Disordered regions are shown in gray color. The PAM region is highlighted in purple dashed rectangles. **e** View of the R-loop structure in the complex. **f** Phylogenetic tree of type V Cas effectors and the ancestral TnpB nuclease based on their structures. **g** Motion vector map showing conformational changes of Cas12h1 induced following target recognition. The surveillance complex model shown as gray cartoon. **h** Structural comparison of the surveillance (up) and interference (bottom) states in surface, showing the structural rearrangements of Helical I and Helical II domain, which expose the RuvC catalytic site (shown in red dashed circle). The PI domain (shown in yellow dashed circle) is invisible in the surveillance state

To investigate how Cas12h1 recognizes, unwinds, and cleaves target DNA, we assembled the Cas12h1^WT^-crRNA-dsDNA ternary complex by incubating the Cas12h1^WT^-crRNA complex with a 26-nt complementary TS DNA and an NTS DNA containing a 5ʹ-ATG-3ʹ PAM sequence in the presence of Mg^2+^, which represents the interference state (Cas12h1^WT^-crRNA-dsDNA, termed the interference complex). The ternary structure was determined using cryo-EM to an average resolution of 3.00 Å (Supplementary Fig. 5). Most of the residues and nucleotides are visible in the interference structure (Fig. 3a–c). Notably, the previously invisible PI domain became stable in the interference state. The previously flexible spacer region of crRNA forms a 17-bp A-form heteroduplex with the complementary TS DNA. The bases dA(-18) to dC(-20) in TS and A(19) to U(26) in crRNA are invisible in the complex. The nucleotides dG(14*) to dA(20*) are invisible in the structure, consistent with the biochemical finding that Cas12h1 cuts the phosphate group between dT(13*) and dG(14*) on the NTS (Fig. 3d, e). A comparison of the surveillance and interference states of Cas12h1 revealed that the Helical I and the Helical II domains underwent substantial conformational rearrangements (Fig. 3g, h).

To investigate the evolutionary relationship between Cas12h1 and other type V Cas effectors, we depicted the phylogenetic tree of Cas12 effectors based on representative structures in RSCB Protein Data Bank (PDB) using the DaLi server^21^ (Fig. 3f). The evolutionary analysis reveals that the closest structural homolog to Cas12h1 is Cas12b. Cas12h1 and Cas12b consist a distinct branch in Cas12 effectors and Cas12h1 represents an earlier evolutionary stage. Each domain of Cas12h1 is more compact than that of Cas12b (Supplementary Fig. 6a), indicating that the proteins increase in size during evolution. In addition to the protein components, the structures of the guide RNAs also display similarities: Cas12h1 crRNA perfectly overlaps with the Stem 3 and R:AR duplex 2 region of the Cas12b crRNA (Supplementary Fig. 6b).

We further investigated the structural similarities between Cas12h1, other Cas12s, and their ancestor, TnpB, by comparing their domain architectures. The Cas12 effectors are characterized by a single RuvC domain responsible for target degradation.^8^ Within the core domains, the RuvC domain displays a typical RNaseH fold, executing target cleavage; the WED domain is characterized by the central β-barrel, playing roles in crRNA and PAM duplex recognition; the Helical domain is consisted of helix bundles, responsible for target recognition; the Nuc domain exhibits the most structural diversity, assisting in target degradation. Evolution has introduced new domains and the expansion of protein properties could have enhanced the stability of the effector complexes within the cellular environments.^9^ The Helical II domain enhances the crRNA-TS interaction at PAM-distal heteroduplex, while the PI domain strengthens the PAM duplex recognition, both contributing to the target specificity required for CIRPSR-Cas adaptive immunity.^22^

### The mechanism of broad-spectrum PAM recognition by Cas12h1

**Fig. 4:**
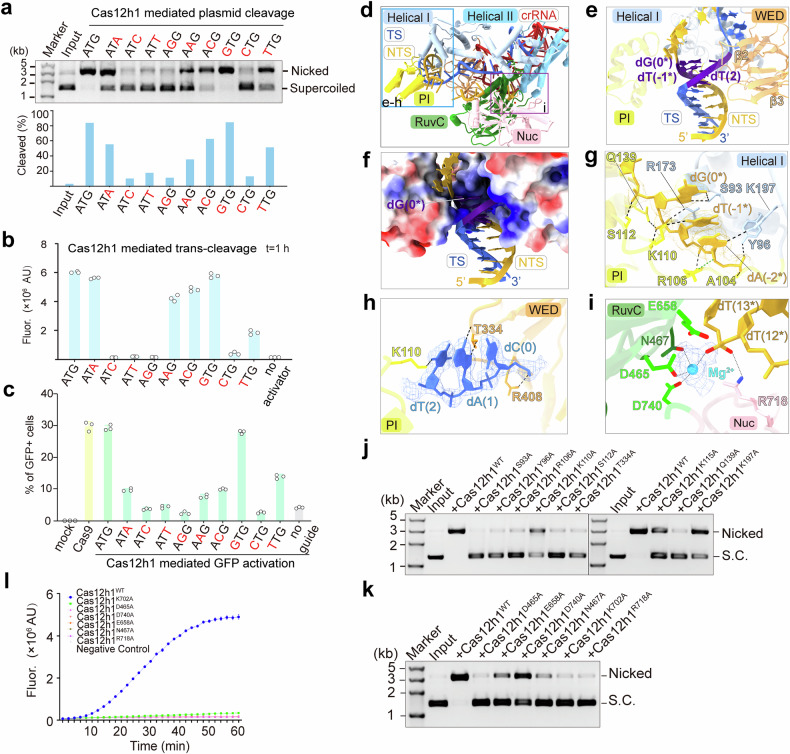
Mechanisms for recognition of a broad-spectrum 5ʹ-DHR-3ʹ PAM and target degradation. **a** Investigation of the PAM recognized by Cas12h1 for *cis*-cleavage. Top, supercoiled plasmid cleavage assays by Cas12h1 with a mutated PAM sequence. The mutated nucleotide is shown in red and the wild-type sequence (5ʹ-ATG-3ʹ) is shown in black. Bottom, the related cleavage efficiency of Cas12h1 challenged with mutated PAM. **b** F-Q assay investigating of the PAM recognized by Cas12h1 for *trans*-cleavage. **c** Identification of the PAM recognized by Cas12h1 with EGFP activation assays in human cells. Cas12h1 was guided by two guides targeting both the sense and antisense strand of VEGF locus with the same designing as in Fig. 1b, except the variation of PAM in both sense and antisense strand. Cas9 was used as a positive control, and Cas12h without a guide RNA was used as a negative control. **d** The relative positions of (**e**–**i**) in the Cas12h1 interference complex. **e** Recognition of the PAM duplex in the Cas12h1 interference complex and the PAM-interacting cleft formed by Helical I, PI, and WED domains. A loop in WED domain contacts the major groove of the PAM duplex. **f** Electrostatic potential surface of the PAM-interacting cleft. **g** Recognition of the NTS DNA in the PAM duplex, with the density of nucleotides shown in mesh. Black dashed lines indicate hydrogen bonds and green dashed line indicates contact. **h** Recognition of the TS DNA in the PAM duplex, with the density of nucleotides shown in mesh. **i** Detailed interactions between the NTS, Mg^2+^ and the RuvC catalytic site of Cas12h1, with the density of Mg^2+^ shown in mesh. **j** Supercoiled plasmid cleavage assay by wildtype Cas12h1 and the mutants in the complex with crRNA. **k** Supercoiled plasmid cleavage testing the *cis*-cleavage activity of wildtype Cas12h1 and its mutants at the catalytic site. **l** F-Q assay comparing the *trans*-cleavage activity of wildtype Cas12h1 and its mutants at the catalytic site

Recognition of a PAM sequence is a prerequisite for target DNA unwinding and R-loop formation.^23^ We assessed the PAM sequence recognized by Cas12h1. Starting with a 5’-ATG-3’ PAM, replacement of dG(0*):dC(0) with dT(0*):dA(0) or dC(0*):dG(0) abolished the DNA cleavage activity, in consistent with the previous studies.^8^ Notably, the substitution of dT(-1*):dA(1) with dA(-1*):dT(1) or dC(-1*):dG(1), or mutating dA(-2*):dT(0) to dG(-2*):dC(0) or dT(-2*):dA(0) retains at least partial DNA cleavage activity, suggesting Cas12h1 recognizes a wider range of PAM than previously report, which is 5’-DHR-3’ (Fig. 4a). Accordingly, a target dsDNA carrying a 5’-DHR-3’ PAM activates the *trans*-activity of Cas12h1 (Fig. 4b). In addition, Cas12h1 recognizes a 5’-DHR-3’ PAM in human cell line (Fig. 4c).

We next sought to determine the structural basis for broad-spectrum PAM recognition. The 5ʹ-ATG-3ʹ PAM duplex is located in a positively charged cleft formed by the PI, WED, and Helical I domains (Fig. 4d–f). A loop between strands β2 and β3 of the WED domain contacts the major groove of the PAM duplex, but no loop was found inserting into the minor groove, unlike its structural homologs, Cas12b^18^ and Cas12i2^19^ (Supplementary Fig. 7a). The PAM duplex is stabilized by intensive interactions between the Cas12h1 protein and the phosphate backbone. Specifically, the side chains of Y96, R173, S112, Q139, and the main chains of A104 and R106 form hydrogen bonds with the NTS phosphate backbone; the side chain of R408 interacts with the TS phosphate backbone (Fig. 4g, h). Alanine substitution of these residues significantly impaired its cleavage ability (Fig. 4j). Meanwhile, several hydrogen bonds contribute to the base-specific recognition of the PAM. In detail, the side chain of T334 forms hydrogen bonds with the N7 and N6 atoms of dA(1). The side chain of K110 from the PI domain forms hydrogen bonds with the O2 atom of dT(2) and the O2 atom of dT(-1*) concurrently. The side chain of S93 forms hydrogen bonds with the O6 and N7 atoms of dG(0*), and the side chain of K197 forms a hydrogen bond with the N7 atom of dA(-2*). Biochemical assays suggested that the T334A and S93A mutation strongly reduced the cleavage activity (Fig. 4j), suggesting the hydrogen bonds between S93, T334 and the bases are important for base-specific recognition.

### Mechanisms of R-loop formation

In the Cas12h1^WT^-crRNA-DNA interference complex, the two-strand substrate DNA molecules unwound beyond the PAM duplex. Several motifs or residues contribute to target unwinding and heteroduplex formation. A loop connecting helix α3 and helix α4 acts as a wedge to favor DNA unwinding and its deletion variant (replaced by residue GGSGGS) slightly reduced the cleavage activity (Supplementary Figs. 7d, e, h). The positively charged residue R408 forms a hydrogen bond with the backbone phosphate group of dC(0), acting as a phosphate lock that stabilizes the heteroduplex, which is also found in other Cas effectors.^17,23–26^ The R408A variant greatly reduced the nuclease activity, further collaborating the importance of the phosphate lock (Supplementary Fig. 7h).

The mature crRNA turns ~180° from the stem duplex of the repeat region at position A(1) (Supplementary Fig. 7f). More specifically, the side chain of R8 forms hydrogen bonds and concurrently stacks with A(1). Biochemical studies showed that the R8A mutation abolished DNase activity (Supplementary Fig. 7h). The NTS is guided through a groove formed by RuvC and the Nuc domain to the catalytic site (Supplementary Fig. 7g).

### The catalytic site of Cas12h1

The RuvC and the Nuc domain form a pocket to accommodate the NTS, and three acidic residues (D465, E658, and D740) in the RuvC domain constitute the catalytic center. Many type V Cas effectors execute target degradation in an Mg^2+^-dependent way and we modeled an Mg^2+^ for the density between the catalytic triad and the target (Fig. 4i). Biochemical assays indicate that Mg^2+^ is critical and sufficient for target degradation of Cas12h1, and combination of Mg^2+^ with Mn^2+^ or Co^2+^ enhances the TS cleavage of Cas12h1, suggesting Mn^2+^ or Co^2+^ may participate in stabilizing the TS for its cleavage (Supplementary Figs. 7i–k). The polar side chains of D465, D740, and N467 interact with Mg^2+^ and may contribute to stabilizing Mg^2+^. The R718 from the Nuc domain, together with Mg^2+^, points towards the phosphate groups between nucleotides dT(12*) and dT(13*), suggesting that this position is a cleavage site in the NTS, which is consistent with the cleavage pattern identified by biochemical assays. The alanine substitution of D465 and E658 greatly reduced the cleavage activity of Cas12h1. Unexpectedly, D740A only caused an approximately half-reduction in Cas12h1 cleavage activity (Fig. 4k), which is different from the critical role of catalytic triads in other type V Cas effectors.^15,16,18–20,26^ In contrast to those of *cis*-cleavage, all the Cas12h1 variants, including D740A, did not exhibit *trans*-cleavage activity (Fig. 4l). These results suggest that although the three conserved catalysts participate in substrate degradation, their roles may differ between *cis*- and *trans*-cleavage.

### Activation of Cas12h1

To investigate how Cas12h1 is activated after target recognition, we reconstituted the second ternary complex, which consists of the Cas12h1 D465A mutant, crRNA, and target DNA in the absence of Mg^2+^; this complex is hereafter referred as the R-loop formation complex (Cas12h1^D465A^-crRNA-dsDNA). We determined the cryo-EM structure of the Cas12h1 R-loop formation complex at an average resolution of 2.76 Å (Supplementary Fig. 8).

**Fig. 5:**
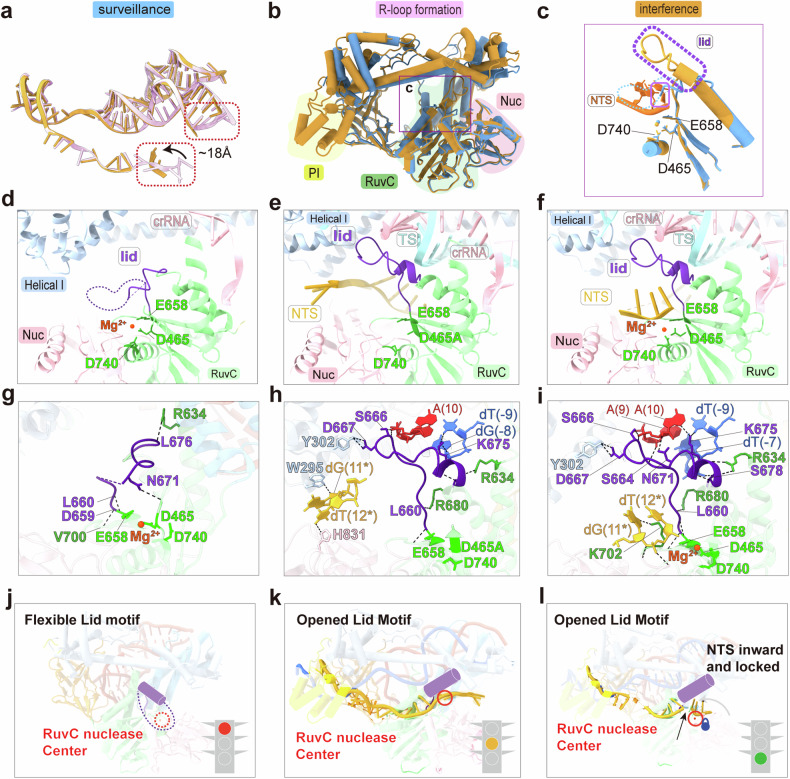
Conformational rearrangements activate Cas12h1. **a** Superimposition of the R-loop structures in the R-loop formation (pink) and interference (orange) state. The comparison between 20 bp heteroduplex in R-loop formation state and 17 bp heteroduplex in interference state and the movement of the NTS DNA was highlighted in red dashed rectangle. **b** The relative positions of (**c**) in the superimposition of the surveillance (blue) and the interference (orange) state. **c** Close-up view of the arrangement of the lid motif in the surveillance (blue) and the interference (orange) state. The flexible lid motif in the surveillance state is indicated in a blue dash line and the lid motif in the interference state is highlighted in purple dashed box. The pyrimidine ring of dT(13*) in NTS of the interference state clashes with the lid motif in the surveillance state, which is highlighted by a purple square. The conformations of the lid motif (colored in purple) and the NTS DNA (colored in dark goldenrod) in the surveillance (**d**), R-loop formation (**e**) and interference state (**f**). The catalytic residues are highlighted in lime. Detail interactions of the lid motif in the surveillance (**g**), R-loop formation (**h**) and interference (**i**) state of Cas12h1. Hydrogen bonds were shown in black dashed lines. A model for the activation of Cas12h1 during surveillance (**j**), R-loop formation (**k**) and interference (**l**) states

Superimposition of the structures shows Cas12h1 undergoes substantial conformational rearrangements from surveillance state to R-loop formation (Supplementary Figs. 9a-e). The NTS moves ~18 Å towards the catalytic site for first strand cleavage (Fig. 5a, Supplementary Fig. 9f). Moreover, the crRNA and the TS DNA form a 20 bp heteroduplex in the R-loop formation state (Fig. 5a), in contrast to the 17 bp heteroduplex in the interference state, indicating that the TS has partially disassociated with the crRNA and been loaded into the catalytic site for second strand cleavage.

Importantly, a loop connecting strand β13 and helix α19 that blocks the catalytic triad and acts as a lid that enables RuvC activation. Although the lid motif (659–678 aa) is flexible in the surveillance state, structural superimposition of the motif suggested that the lid motif in the surveillance state clashes with the NTS in interference state at dT(13*), indicating that the lid motif sterically blocks the substrate from the catalytic site before target recognition (Fig. 5b, c). A comparison of the conformational changes in the lid motif revealed that in the surveillance state, the lid motif was mostly highly flexible (662–670 aa invisible; Fig. 5d); then, the crRNA:TS heteroduplex helps turn up the lid motif and stabilize the lid in an “open” conformation where the catalytic site is exposed (Fig. 5e); finally, the NTS turned inwards and was loaded to the catalytic site for cleavage (Fig. 5f). Specifically, the flexible lid motif is kept in an “off” conformation through hydrogen bonds between L660-E658, D659-V700, N671-L466, and L676-R634 (Fig. 5g). After target recognition, the lid motif forms new hydrogen bonds with the heteroduplex (S666-A(10), K675-dT(-9), K675-dG(-8)), together with other new hydrogen bonds between D667-Y302, L676-R634, and L660-R680, which stabilize and keep the lid motif in an “open” conformation (Fig. 5h). Finally, the NTS turns inwards, and additional hydrogen bonds between the lid motif and the heteroduplex (S664-A(9), N671-A(10), S678-dT(-7)) are generated, further stabilizing the lid motif to favor substrate cleavage (Fig. 5i). Replacement of the lid motif (659–678 aa) by a short glycine-serine linker (GGS) abolished nuclease ability of Cas12h1 (Supplementary Fig. 7l). Notably, K702 functions as a lock that stabilizes the NTS for degradation (Fig. 5i). Biochemical studies revealed that the K702A mutation abolished both the *cis*- and *trans*-nuclease activities (Fig. 4k, l). Based on these structural discoveries we summarize a model for the catalytic mechanism of Cas12h1 (Figs, 5j–l, Supplementary Fig. 11).

### Development of Cas12h1 as a nucleic acid detector

**Fig. 6:**
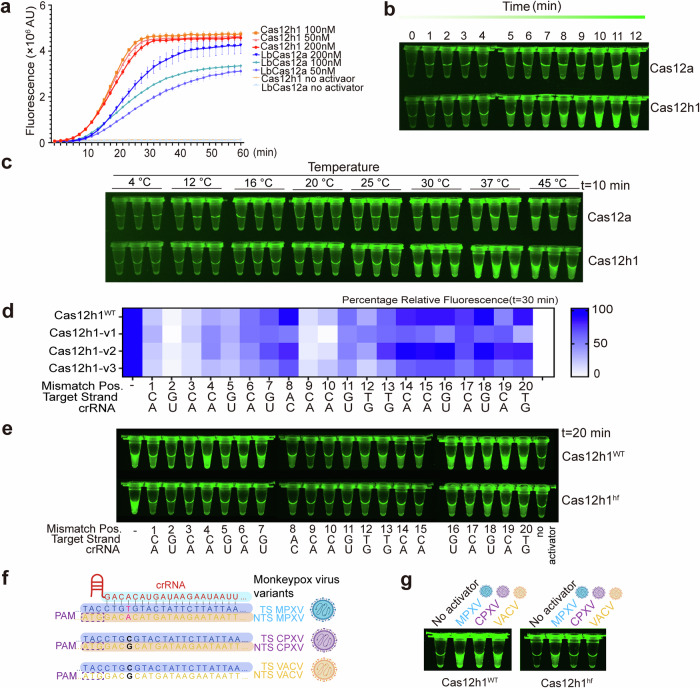
Development of Cas12h1 as a nucleic acid detector with rationally engineered Cas12h1 variants. **a** F-Q assay comparing Cas12h1 and LbCas12a when challenged with a 40 bp dsDNA activator at various concentrations. **b** Visual detection of fluorescence readout comparing the time-course *trans*-cleavage activity of Cas12h1 and LbCas12a. **c** Visual detection of fluorescence readout comparing *trans*-cleavage activity of Cas12h1 and LbCas12a at different temperatures. **d** Mismatch F-Q assay for detection of a 40 bp dsDNA activator with a 5’-ATG-3’ PAM for wildtype and Cas12h1 variants (*n* = 3 independent replicates; mean ± sd). Only one-point data at time = 30 min is shown. **e** Visual detection of fluorescence readout comparing the specificity of Cas12h1^WT^ and Cas12h^hf^ with single nucleotide mismatch dsDNA activators. **f** Schematic representation of the target sequences of the Monkeypox virus (MPXV), Cowpox virus (CPXV), and Vaccinia virus (VACV), with the different nucleotide highlighted in pink. **g** Visual detection of fluorescence readout comparing the specificity of Cas12h1^WT^ and Cas12h^hf^ when detecting the MPXV (on-target substrate), or CPXV/VACV (mismatch substrate)

The *trans*-cleavage of type V Cas effectors enables them to detect DNA. Cas12a has been harnessed as a nucleic acid diagnostic tool for pathogen detection,^11,27^ SNP genotyping^28^ and cancer screening.^29^ To explore the potential of Cas12h1 as a nucleic acid detector, we compared the *trans*-cleavage efficiency of Cas12h1 with that of the widely used LbCas12a. Cas12h1 generated more significant fluorescence than LbCas12a in a shorter time both in F-Q assays and visual detections (Fig. 6a, b). Notably, Cas12h1 generated visible fluorescence at room temperature (25 °C) after 10 minutes (Fig. 6c), and the fluorescence became more obvious as the temperature increased. These results suggest that Cas12 h1 is a promising equipment-free, time-saving nucleic acid detector.

We next attempted to improve Cas12h1 through protein engineering, since Cas12h1 exhibited single nucleotide mismatch tolerance in *trans* (Fig. 2h). We hypothesized that the substitution of negatively charged residues that interact with nucleic acids with neutral residues might increase DNA-binding affinity and structural stability.^12,30^ So, we introduced multiple alanine substituents into the negatively charged tips in helix α9, which contacts the TS at the PAM-proximal region (Cas12h1-v1, Supplementary Figs. 7d, m). In addition, a previously-flexible loop between helices α17 and α18 contacts the PAM-distal heteroduplex; therefore, we deduced that this loop might play a role in stabilizing the R-loop in the PAM-distal region. Similarly, we replaced the negatively charged residues in the loop with alanine (Cas12h1-v2, Supplementary Figs, 7d, 7n). Moreover, we mutated the positively charged residue lysine in the loop to arginine to enhance the protein-nucleic acid interaction (Cas12h1-v3, Supplementary Fig 7o). We then tested whether Cas12h1 variants exhibit less tolerance to single-mismatch substrates. A mismatch F-Q assay showed that the fluorescence ratio of Cas12h-v3 single-mismatch/no-mismatch strains was lower than that of wildtype Cas12h1 while retains comparable kinetics (Fig. 6d, Supplementary Fig. 2g). Thus, we renamed Cas12h-v3 as Cas12h1^hf^. Accordingly, Cas12h1^hf^ barely generated visible fluorescence when challenged with a single mismatch at positions 1–12 (Fig. 6e).

Next, we investigated the specificity of Cas12h1^hf^ with a bona fide target. The genus *Orthopoxvirus* includes species pathogenic to humans, such as Monkeypox virus (MPXV), Cowpox virus (CPXV) and Vaccinia virus (VACV). Sharing high nucleotide sequence similarity, it is difficult to distinguish these viruses. To test whether Cas12h1^hf^ could distinguish *Orthopoxvirus* species, we prepared a Cas12h1-crRNA complex targeting the J2R gene on MPXV, which is different from that in CPXV or VACV only in position 4 (Fig. 6f). Accordingly, Cas12h1^hf^ generated comparable fluorescence in MPXV, but barely generated fluorescence with CPXV and VACV (Fig. 6g), suggesting that Cas12h1^hf^ distinguishes MPXV from other *Orthopoxvirus*. Taken together, these findings indicate that the variant Cas12h1^hf^ is a highly specific nucleic detector with single-base resolution.

## Discussion

In this study, we systematically explored the biochemical characterization of Cas12h1, elucidated the underlying molecular mechanisms, and ultimately, rationally engineered Cas12h1 into a highly specific nucleic acid detector, Cas12h1^hf^. In contrast to most Cas12 effectors, which generate dsDNA breaks, Cas12h1 preferentially acts as a nickase and exhibits weak ability to cut the TS DNA. Similar phenomena were observed for the Cas12h1 structural homologs Cas12b^12^ and Cas12i,^8^ but for Cas12h1, the TS cleavage ability seems to be even weaker, since little dsDNA cleavage product was observed only in extreme condition. A possible mechanism for its nickase activity is that Cas12h1 exhibits less protein-nucleotides interactions at the PAM-distal heteroduplex, indicating that Cas12h1 lacks a strong motif that twists the TS toward the catalytic site. This is a distinct mechanism from the nickase variant AsCas12a (R1226A),^31^ whose conserved arginine acts as a stabilizer for the TS cleavage (Supplementary Fig. 12a). In addition, Cas12h1 exhibits nickase activity in human cells, indicating that it follows the same manner in vivo. As a genome editing tool, CRISPR-Cas might lead to undesired outcomes caused by dsDNA breaks.^6^ Alternative strategies, such as prime editing, which utilize the nickase variants of Cas9^3^ and Cas12a^32^ effector, have been developed. The preferential nickase activity of Cas12h1 provides a new candidate nickase tool for genome editing with small size and wide range of PAM recognition.

Structural and phylogenetic analyses offer valuable insights into the evolutionary trajectory of Cas12h1. Although Cas12h1 is identified from metagenomic data with unknown biological origin, analysis of V-H loci shows that Cas12h1 lacks the adaption module, whose features are shared by other subtypes discovered from phage genome (e.g., Cas12j and Casλ), indicating phages are possible biological origins of Cas12h1 (Supplementary Fig. 6c). Probably phages invaded bacteria harboring ancient CRISPR-Cas12b system, hijacked ancient Cas12b and developed into Cas12h1. Sharing a common ancestor, Cas12b have evolved to a relative larger size to achieve more efficient immune activities, while Cas12h1 retains the core region of the protein and guide RNA, possibly to keep a compact size suitable for a small viral genome size. Cas12h1 and Cas12b provide hints for a possible co-evolution mechanism for CRISPR-Cas system within phages and hosts.

The recognition of the PAM sequence is a prerequisite for target unwinding and cleavage. The type V Cas proteins preferentially recognize a T-rich PAM sequence.^8,13,33–39^ In contrast, Cas12h1 recognizes a wide range of PAM sequences, 5ʹ-DHR-3ʹ. The type V Cas effectors adopt both base-specific and shape-specific strategies to recognize PAM sequences.^15,40^ Structural and biochemical assays suggested the hydrogen bonds within S93-Cas12h1 and T334-Cas12h1 are important for base-specific recognition. No hydrogen bond exists between T334 and the TS when we modeled dG(-1*):dC(1) to replace dT(-1*):dA(1), which is the possible reason why the second nucleotide is “non-G” (Supplementary Fig. 7b). In addition, the hydrogen bond between the N7 atom of dG(0*) and S93 was retained when we mutated dG(0*) to dA(0*), while no hydrogen bond exist between S93 and dC(0*) or dT(0*), which explains why the third nucleotide in the PAM sequence is R (Supplementary Fig. 7c). For shape-specific recognition, the structural homologs of Cas12h1 (Cas12b and Cas12i) recognize their PAM sequence through a loop inserting the minor groove of the PAM region. Similarly, Cas12a recognizes its PAM in a sequence-independent way through a helix contacting the minor groove in PAM region.^15^ However, there is no motif in Cas12h1 that contacting the minor groove in the PAM. The less contacts between Cas12h1 and the minor groove of the PAM duplex indicate that Cas12h1 may not recognize the PAM sequence in a sequence-independent manner, and thus relaxes the requirement for PAM sequence. The stringent PAM requirement narrows target selection and limits precision genome editing applications. The structural basis of the broad-spectrum Cas12h1 may provide clues for developing new “PAMless” Cas variants for various applications.

Structural studies suggest that nuclease activation by Cas12h1 occurs in a similar but distinct manner. Many type V Cas effectors share a conserved mechanism for the RuvC activation, where a structurally conserved lid undergoes a loop-helix or helix-loop transition and rotates up during R-loop formation to expose the catalytic site (Supplementary Fig. 12b).^20,41,42^ In contrast to the pre-ordered lid motifs in other Cas12 effectors, Cas12h1 possesses a flexible lid in the surveillance state; the lid motif undergoes a “flexible-to-stable” transition during nuclease activation and rotates upwards to expose the catalytic site. The formation of the crRNA:TS hybrid triggers conformational rearrangement of the Helical I and Helical II domains, stabilizes the flexible lid motif and initiates target cleavage. The distinct activation manner in Cas12h1 indicates the diverse activation mechanisms in the type V Cas effectors.

Efforts have been made to improve the sensitivity and specificity of Cas effector detection. We rationally engineered a Cas12h1 variant, Cas12h1^hf^, which is more sensitive to single-base mismatches than is the wild-type Cas12h1. A variety of high-fidelity Cas9 variants have been developed based on different strategies, such as minimizing nonspecific interactions with targets,^43^ weakening the NTS binding,^44^ and deleting the motif that participates in stabilizing the mismatch structure.^7^ Structural studies of mismatches in Cas9 suggest that off-target binding is enabled by noncanonical base pairing interactions and accommodated solely by conformational distortion of the TS DNA within the seed region because of the structural rigidity of the gRNA. This increases the energetic penalty of base mispairing, which is the reason why there is less tolerance of mismatches within the seed region.^45^ In Cas12h1^hf^, the lysine residues in the loop are replaced by arginine, which possesses a longer side chain and stronger positive charge, increasing engagement with PAM-distal heteroduplex, as well as the energy required for mismatch tolerance; thus, Cas12h1^hf^ is less tolerant of single-base mismatches. Together with its wide-range temperature adaptability and fast cleavage kinetics, Cas12h1^hf^ is a promising diagnostic tool suitable for point-of-care detection of pathogens and other nucleic acids. In summary, the discover of the new nickase available for genome editing, the elucidation of underlying molecular mechanisms, and the development of high-fidelity nucleic acid detector improve the understanding of Cas12 effectors, expand the CRISPR toolbox and provide implications for developing more reliable genome editing and nucleic acid diagnostic tools.

## Materials and methods

### Protein expression and purification

The heterogenous expression and purification of Cas12h1-crRNA complex were performed as previously described.^26^ In brief, the pET-30b vector encoding Cas12h1 or its variants and pCDFDuet-1 vector encoding the CRIPSR array were co-transformed into *E. coli* Rosetta (DE3) cells (Novagen). The expression was induced by 0.2 mM IPTG at 18°C overnight. The cells were harvested and lysed by sonication in Buffer A (25 mM Tris-HCl, pH 7.5, 500 mM NaCl, 3.5 mM β-mercaptoethanol, 1 mM PMSF). The supernatant was loaded on a Ni-NTA (QIAGEN) and eluted with Buffer B (25 mM Tris-HCl, pH 7.5, 350 mM NaCl, 2 mM MgCl_2_, 2 mM DTT, 200 mM imidazole), followed by a heparin column (GE) and eluted with a linear sodium chloride gradient, and then by size exclusion chromatography (Superdex 200, GE) in Buffer D (Buffer B without imidazole). Peak fractions were concentrated and stocked in −80 °C. For preparation of ternary complex, the Cas12h1-crRNA complex was incubated with annealed target duplex at a 1:1.2 molar ration in Buffer B and incubating at 16 °C for 1 h, followed by size exclusion chromatography.

### Cryo-EM data collection and processing

The complexes were applied to Au 300-mesh R1.2/1.3 grids (Quantifoil) in a Vitrobot Mark IV plunger (Thermo Fisher Scientific). The excess liquid was removed at a blot force of 6 s at a blot force of –3 at 4 °C, and 100% humidity and then plunge-frozen in liquid ethane, cooled with liquid nitrogen. The grids were plunge-frozen in liquid ethane, cooled with liquid nitrogen. The cryo-EM data were collected on a 30-kV Titan Krios microscope (Thermo Fisher Scientific) equipped with a Gatan K3 Summit direct detector and Gatan BioQuantum energy filter. Data were acquired using an EPU software at nominal magnification of ×105,000 (0.855-Å size) with a defocus range of −1.2 to −1.8 µm in counting mode.

For Cas12h1 complexes, the 32 frames (the first 3 frames skipped) of each image stack in super-resolution model were aligned, decimated, summed and dose-weighted using MotionCor2. Then, CTF values of the summed micrographs were determined using CTFFIND-4.1. The following 2D classification, ab initio reconstruction and hetero refinement were completed in cryoSPARC. Local resolution was calculated with the half maps in RELION-3.1. More detailed information be seen in Supplementary Table 1 and Supplementary Figs. 3, 5, 7.

### Model building and refinement

The constructed model of Cas12h1 complexes were building using Coot, followed by refinement with Phenix.real_space_refine. Molecular visualization figures were prepared with PyMol (http://pymol.org) and ChimeraX (https://www.cgl.ucsf.edu).

### In vitro cleavage assays

For supercoil plasmid cleavage assays, a typical cleavage reaction contains 1 μg substrate DNA, 200 nM wildtype or mutant Cas12h1-crRNA complex in 1× FastDigest Buffer (Thermo Scientific™) at a volume of 30 μL. The reaction was carried out at 37 °C for 30 min and quenched by adding 100 mM EDTA and 8 mg/mL Proteinase K. For oligonucleotide cleavage assay, the fluorescence-labeled oligos were synthesized from Sangon Biotech (Shanghai, China). A typical assay contains 2 μM target, 4 μM Cas12h1-crRNA complex in 1× FastDigest Buffer at 37 °C for 30 min, then quenched by 100 mM EDTA and 8 mg/mL Proteinase K.

### Fluorophore-quencher assay

A typical F-Q reaction contains 200 nM Cas12h1-crRNA complex, 500 nM reporter, and 100 nM 40-bp-dsDNA activators in 1 × FastDigest Buffer with total volume adjust to 20 μL and the reactions were carried at 37 °C. The assays were performed in a 96-well flat-bottom polystyrene assay plate. For visual detection, the reactions were terminated by 100 mM EDTA and the results were read under ultraviolet light.

## Supplementary information

Supplemntary Files

## Data Availability

Cryo-EM reconstructions of Cas12h1-crRNA surveillance complex, Cas12h1^D465A^-crRNA-dsDNA R-loop formation complex, and Cas12h1^WT^-crRNA-dsDNA interference complex have been deposited in the Electron Microscopy Data Bank with codes EMD-39082, EMD-39083, and EMD-39084, respectively. The atomic coordinates have been deposited in the Protein Data Bank with the accession codes 8Y9L, 8Y9M, and 8Y9N, respectively. Other data are available from the corresponding author upon reasonable request.
