# Supplementary material for: Molecular insights and rational engineering of a compact CRISPR-Cas effector Cas12h1 with a broad-spectrum PAM
Source: Signal Transduct Target Ther. 2025 Feb 12;10:66. doi: 10.1038/s41392-025-02147-5 (PMC11830025; doi:10.1038/s41392-025-02147-5)

Supplementary Materials for

Molecular insights and rational engineering of a compact CRISPR-Cas effector Cas12h1 with a broad-spectrum PAM

Weiwei Zheng^1,3^, Hongyu Li^1,3^, Mengxi Liu^1,3^, Yuhang Wei^1,3^, Bo Liu^1^, Zekai Li^1^,Chenyang Xiong^1^, Shiqing Huang^1^, Chunyi Hu^2,#^ and Songying Ouyang^1,4,#^

Correspondence to: [huchunyi@sibcb.ac.cn](mailto:huchunyi@sibcb.ac.cn), [ouyangsy@fjnu.edu.cn](mailto:ouyangsy@fjnu.edu.cn)

**This PDF file includes:**

Supplementary Methods and Meterials

Supplementary Figures. 1 to 12

Supplementary Tables 1 to 3

**Other Supplementary Materials for this manuscript include the following:**

Uncropped agarose gel analysis and western blots

Supplementary Methods and Materials

**Plasmid construction**

For protein heterogenous expression and purification, the *E.coli*-codon-optimized Cas12h1 gene with 6×His tag was cloned into the pET-30b vector between the *Nde*I and *Xho*I sites. The Cas12h1 CRISPR array template was synthesized by General Biotech (Anhui, China) and cloned into the *Nde*I and *Xho*I sites of the pCDFDuet-1 vector. The LbCas12a gene was synthesized and cloned into the pET-28a vector. For *in vitro* cleavage assay, the plasmid DNA substrates was formed by annealing the coding oligonucleotide strand and inserting it between the *Xba*I and *Bam*HI sites of the pUC-19 vector. For Cas12h1 variants, the PCR reactions were carried out to accomplish the site-directed mutagenesis using wildtype Cas12h1 as template. The results were confirmed by Sanger-sequencing.

**Protein expression and purification**

For the heterogenous expression and purification of LbCas12a-crRNA complex, the pET-28a encoding LbCas12a was transformed into the *E. coli* Rosetta (DE3) cells and then induced and purified following the same procedure as Cas12h1-crRNA complex. The LbCas12a crRNA was synthesized by General Biotech (Anhui, China) and annealed from 75 ℃. The purified LbCas12a was incubated with crRNA at a ratio of 1:1.2 at 4 ℃, and the complex was then flash-frozen in liquid nitrogen and stored at a temperature of -80 °C for preservation.

***In vitro* cleavage assays**

For temperature variation assay, a total of 1.5 μg substrate was cleaved by 250 nM Cas12h1-crRNA complex for 30 min at indicated temperatures. For S1 nuclease treatment, the cleavage product of supercoiled plasmid, linearized plasmid, or PCR fragment by Cas12h1 was gel-purified and then treated with S1 nuclease (Thermo Scientific™) according to manufactures’ instructions. For Sanger sequencing testing the cleavage product of Cas12h1, the cleavage product was gel-purified for sequencing. For metal ion assays, 200 nM Cas12h1 binary complex was incubated with 10 mM EDTA in 1 × FastDigest Buffer at 4 °C for 2 hours, then the metal ions were added into the reaction at indicated concentrations and a typical cleavage reaction was performed. The cleavage products were analyzed in 1% agarose gel in 1× TAE running buffer or otherwise indicated.

For the *trans*-cleavage activation assay in oligonucleotide cleavage assay, the dsDNA activators containing 11-16 bp sequences after PAM duplex or 11-16 nt ssDNA activators were tested for their ability to trigger the DNase of Cas12h1. A 50 nt non-specific ssDNA carrying a 5’-6FAM fluorescent label was used as substrate for *trans*-cleavage. The reactions contain 3 μM Cas12h1-crRNA complex, 2 μM activators and 2 μM substrate in 1 × FastDigest Buffer.

**Cell culture and EGFP activation assay.**

The HEK293T cells were cultured in DMEM medium with 10% FBS at 37℃ in 5% CO_2_. The cells were seeded in a 24-well plates a day before transfection and transfected when the cell confluency reached 70% in the next day. A total of 1μg plasmid (500 ng GFP plasmid and 500 ng plasmid expressing Cas12h1 and guide RNA) was transfected into HEK293T cells using Lipofectamine 3000 (Life Technologies). When the cells were co-transfected with two guides, the cells were transfected with 500 ng GFP plasmid, 250 ng Cas12h1-guide1 plasmid and 250 ng Cas12h1-guide 2 plasmid. The cells were harvested 48 h after transfection and analyzed for FACS using AriaIII instrument (BD Biosciences).

**Statistics**

All the bars in graphs were depicted using GraphPad Prism (Version 8) and shown as mean±SEM. The experimental findings were independently replicated at least three times.

Supplementary Figure 1.


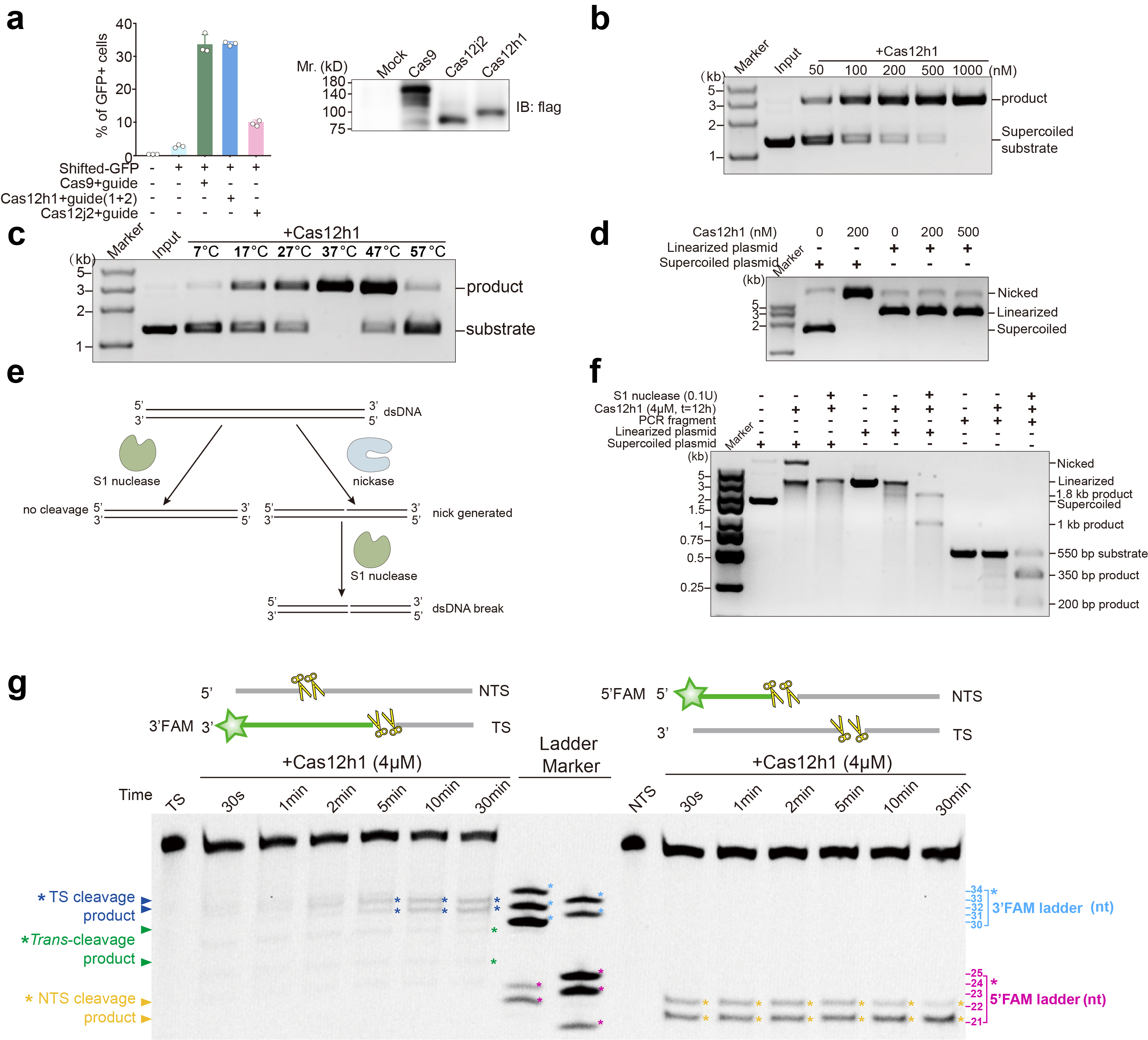


**Supplementary Figure 1. Cas12h1 predominantly cleave the NTS DNA, relating to Figure 1.a,** Comparison of EGFP activation efficiency. Left: EGFP activation efficiencies by Cas9, Cas12h1 and Cas12j2 with their optimal PAM by flow cytometry (5’-ATG-3’ for Cas12h1, 5’-TGG-3’ for Cas9, 5’-TTG-3’ for Cas12j2). The experiment was conducted following the scheme in **Fig. 1b**. Data represents mean ± s.d. of three biological replicates. Right: Western blotting analysis testing the protein expression level. **b,** Supercoiled plasmid cleavage assay testing Cas12h1 DNase activity at different Cas12h1-crRNA complex concentrations. Cas12h1, Cas12h1-crRNA binary complex. The substrate was 1.8μg. **c**, Cleavage of supercoiled plasmid by Cas12h-crRNA complex at different temperatures. A total of 1.5 μg substrate was cleaved by 250 nM Cas12h1-crRNA complex for 30 min. **d,** Linearized plasmid cleavage assay testing Cas12h1 DNase activity. The supercoiled plasmid and cleavage product of supercoiled plasmid by Cas12h1 were used as controls indicating their mobility. **e,** Scheme illustrating the function of S1 nuclease of transferring nicks to dsDNA breaks. **f,** The tests of Cas12h1 DNase activity on supercoiled plasmids, linearized plasmids or a PCR fragment at a high concentration for a long time. The S1 nuclease was introduced after treatment of Cas12h1 to indicate the product of dsDNA breaks. **g,** Representative dsDNA cleavage pattern generated by Cas12h1. Top, schematic representation of the fluorescently labelled target duplex used in the cleavage assays. Bottom, denaturing gel demonstrating the time-course cleavage of the fluorescently labelled target duplex by Cas12h1-crRNA complex. The *cis*-cleavages of the NTS and TS are labelled with the yellow and blue triangles, respectively, and the *trans*-cleavages are labelled with the green triangles.

Supplementary Figure 2.


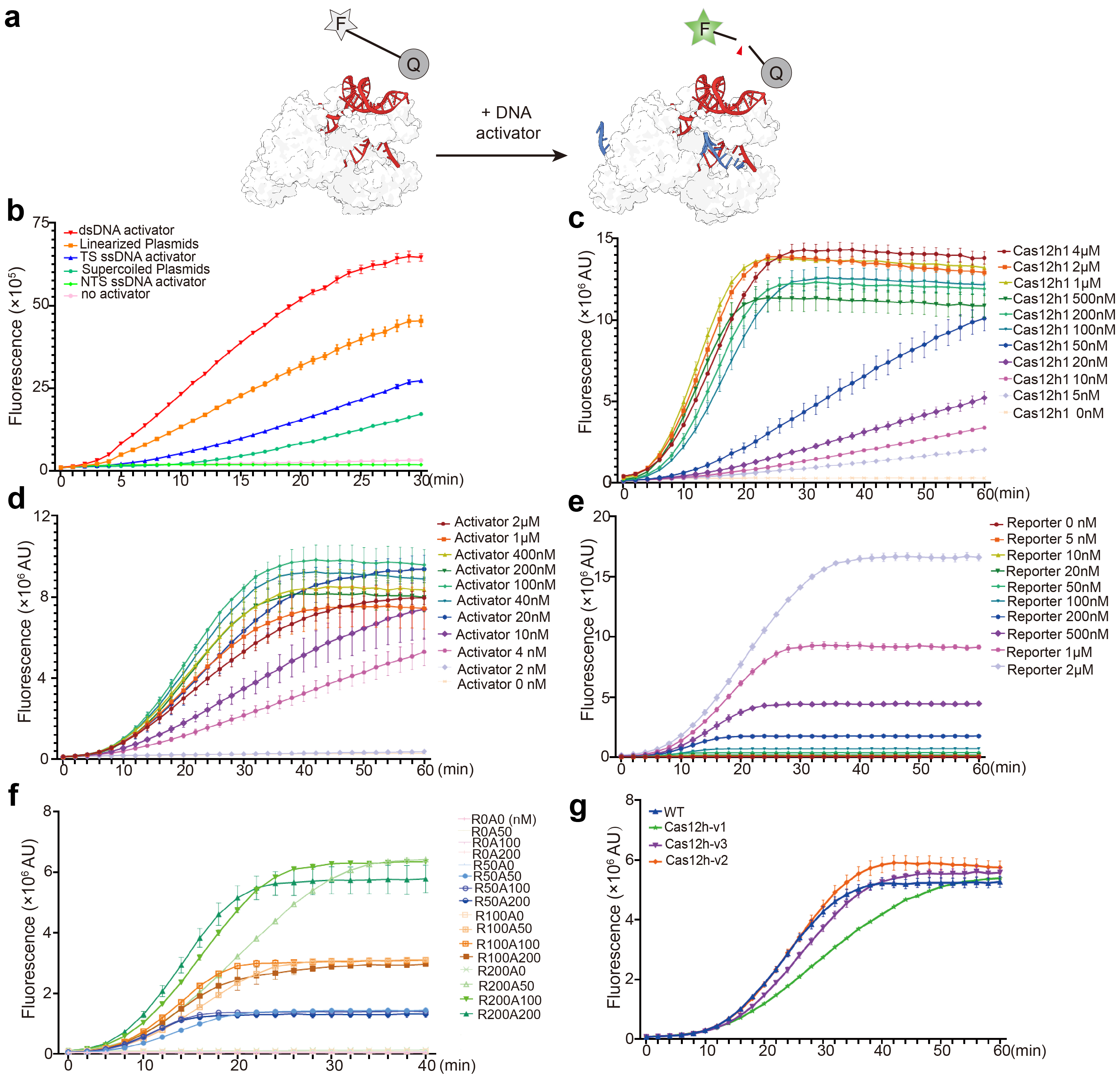


**Supplementary Figure 2. Factors affecting the *trans*-cleavage activity of Cas12h1, relating to Figure 1 and Figure 6. a,** Scheme illustrating the in vitro nucleic acid detection through F-Q assay. **b**, F-Q assay for detection of Cas12h1-catalyaed ssDNA reporter *trans*-cleavage using different DNA activators: a 40 nt TS ssDNA activator complementary to the spacer of crRNA, a 40 nt NTS ssDNA activator complementary to the TS DNA, a 40 bp dsDNA activator annealed from TS and NTS ssDNA, a supercoil plasmid activator mentioned above, and a linearized plasmid activator resulting from SspI treatment. **c,** Different Cas12h1 concentrations, with reporters at 500 nM and activators at 100 nM. **d,** Different DNA activator concentrations, with Cas12h1 at 200 nM and reporters at 500 nM. **e,** Different ssDNA reporter concentrations, with Cas12h1 at 200 nM and activators at 100 nM. **f,** Different reporter and activator combinations. The concentration of Cas12h1 is 200 nM. R, reporters; A, activators. **g**, The *trans*-cleavage activities of wildtype Cas12h1 and its variants. The wildtype Cas12h1 or its variants were activated by a 40 bp dsDNA activator with a 5’-ATG-3’ PAM at a concentration of 100 nM.

Supplementary Figure 3.

**
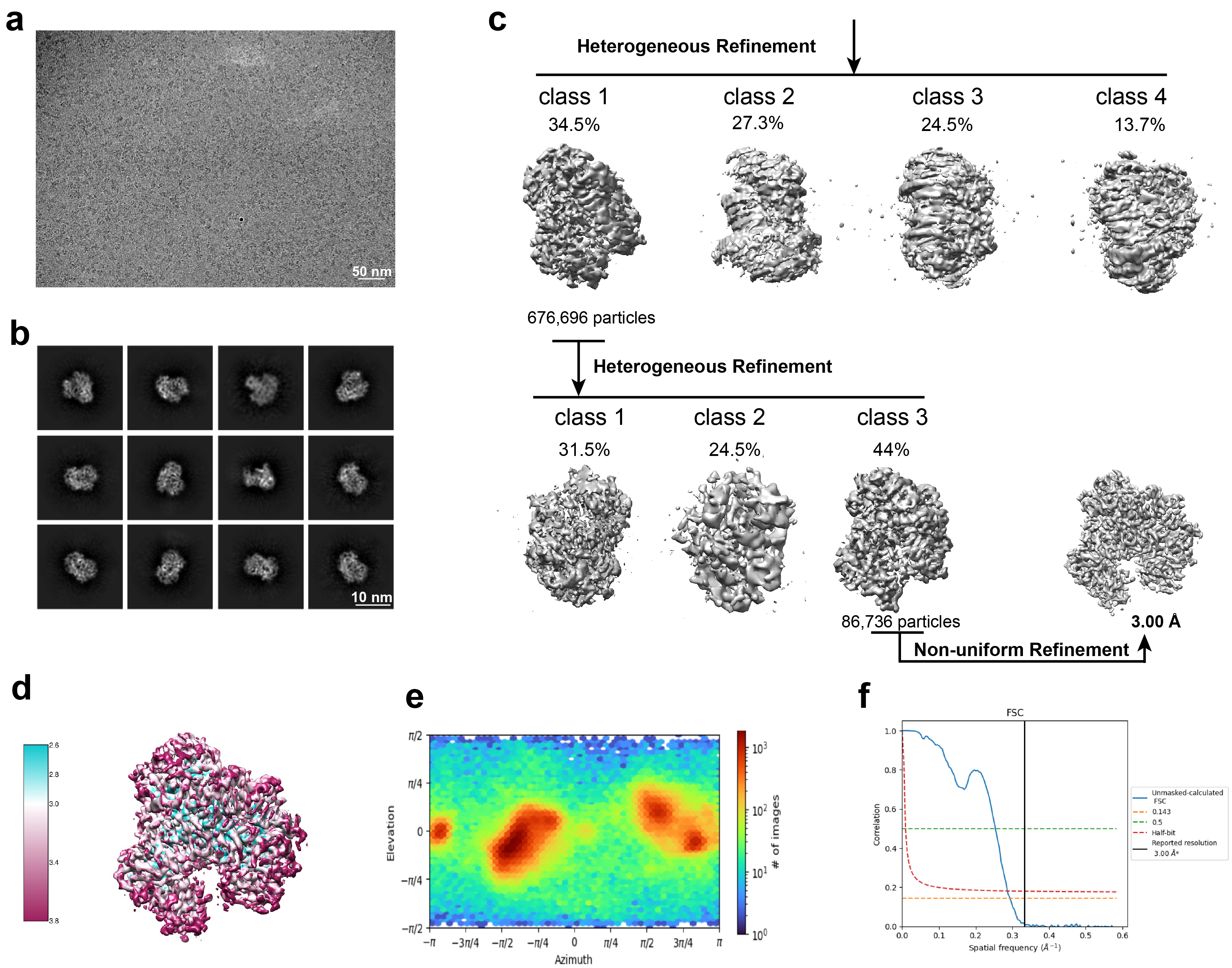
**

**Supplementary Figure 3. Cryo-EM Data Collection, Analysis, and Modeling of the Cas12h1-crRNA surveillance complex, the Cas12h1^WT^-crRNA-dsDNA interference complex and the Cas12h1^D465A^-crRNA-dsDNA R-loop formation complex.**  **a,** A representative raw cryo-EM micrograph of the surveillance complex. **b,** Representative 2D class averages. **c,** Classification strategy and refined maps of the surveillance complex. About 4.4 million particles were auto-picked, and after 2D classification, a total of 679,696 particles for further 3D classification. The homogeneous refinement with 86,736 particles from best class was performed, resulting in a map at 3.00 Å resolution. **d,** Local resolution map for the reconstruction in (**c**). **e** Euler plot for the surveillance complex. **f**, Plot of the global half-map FSC (solid red line). FSC plot for the reconstruction suggests an average resolution of 3.00 Å.

Supplementary Figure 4.


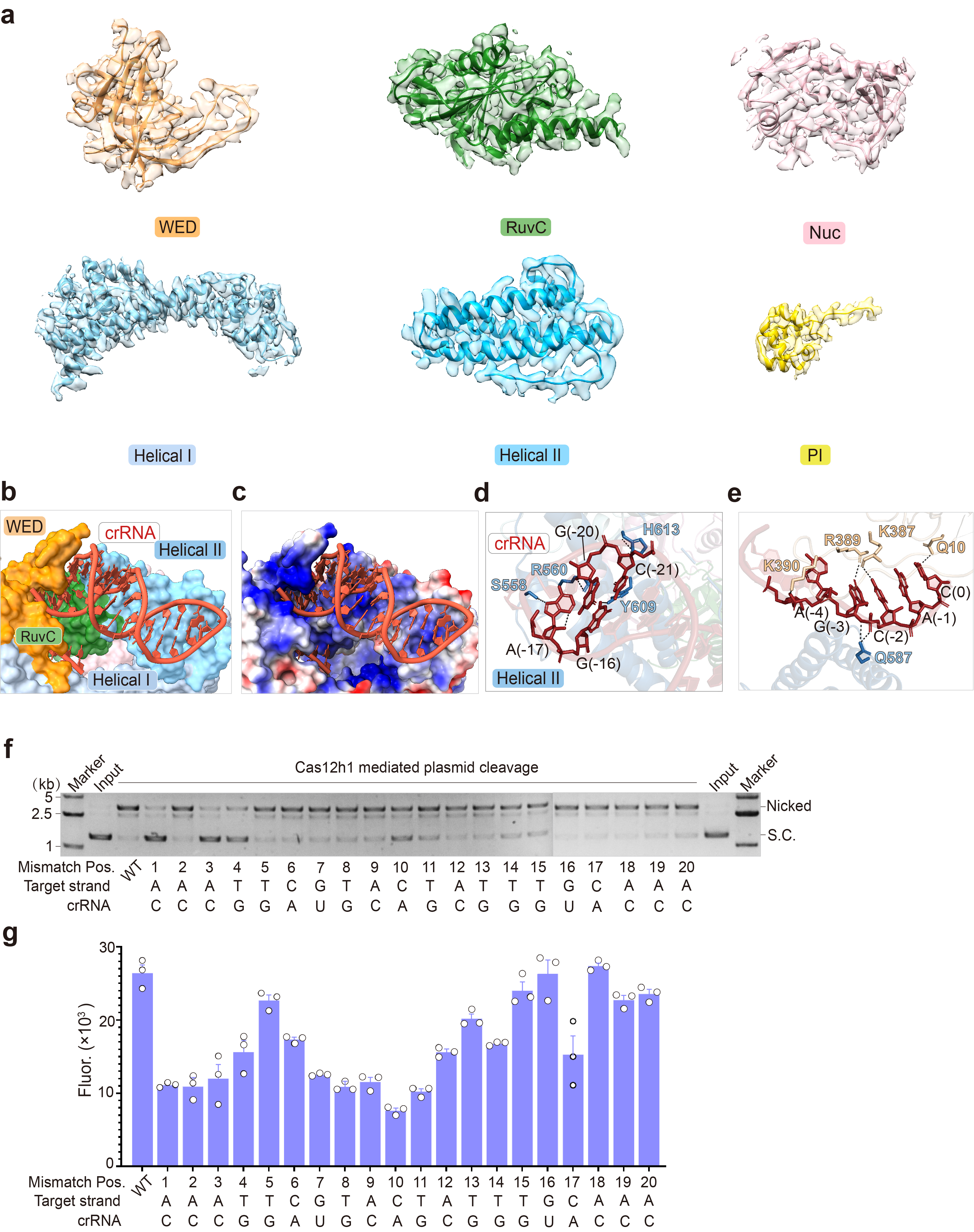


**Supplementary Figure 4. Recognition of the crRNA repeat region. a,** Detailed cryo-EM density map of each domain of Cas12h1 with final models fitted in**.** The cryo-EM density map is shown as transparent surface. **b,** The stem-loop structure of the crRNA repeat region held by the WED, Helical II and RuvC domains in the Cas12h1^WT^-crRNA surveillance complex. **c**, Electrostatic potential surface of the crRNA binding region in the Cas12h1^WT^-crRNA surveillance complex. Red, white, and blue indicate negative, neutral, and positive electrostatic potential surfaces, respectively. **d,** Detailed interactions between the nucleotides C(-21)–G(-16) of the crRNA repeat region and Cas12h1. **e**, Detailed interactions between the nucleotides A(-4)–C(0) of the crRNA repeat region and Cas12h1. **f**, Supercoiled plasmid cleavage testing the *cis*-cleavage activity of Cas12h1 with single mutated target DNA. **g**, Mismatch F-Q assay detecting the *trans*-cleavage activity of Cas12h1 with single mutated target dsDNA activators with a 5’-ATG-3’ PAM.

Supplementary Figure 5.


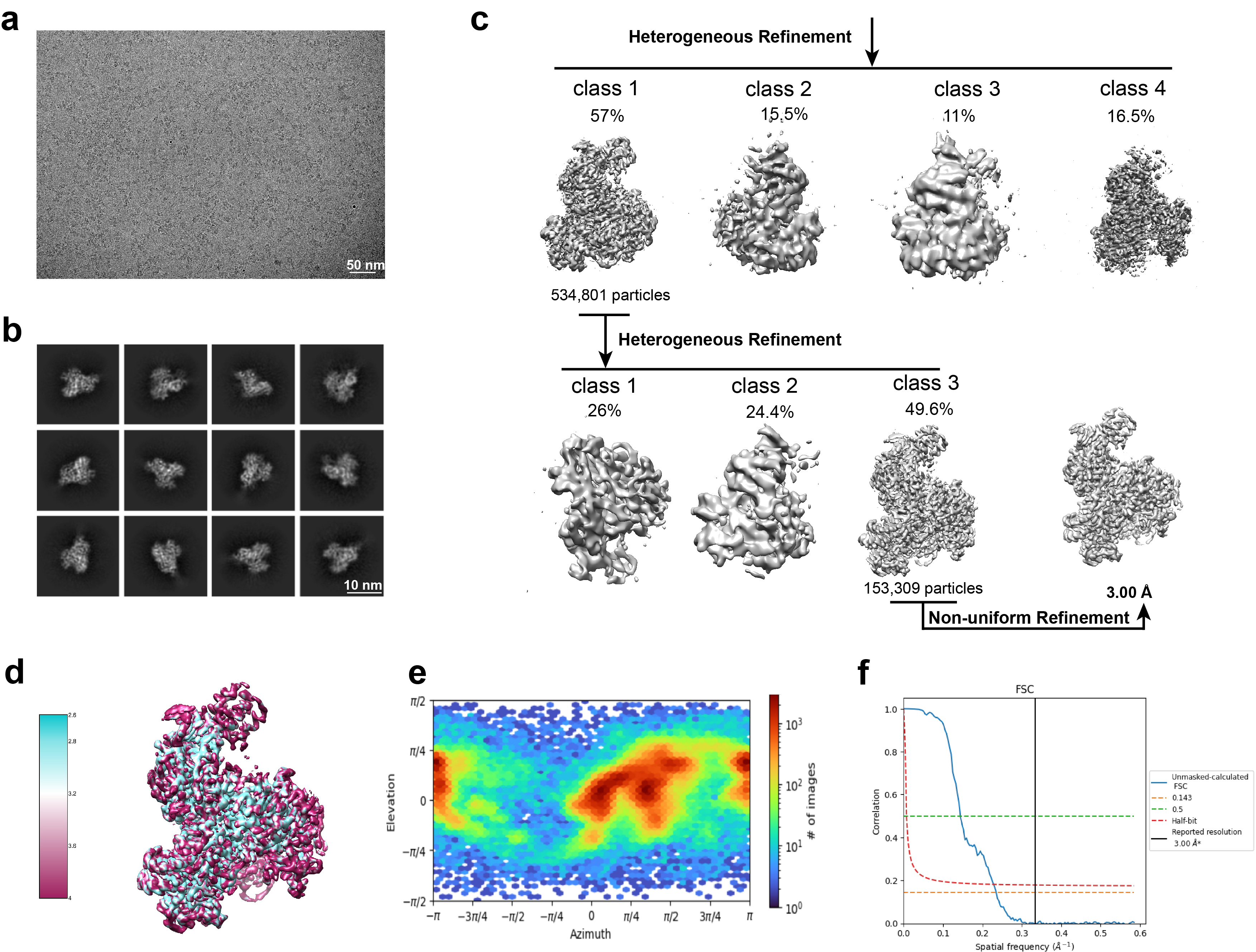


**Supplementary Figure 5. Cryo-EM Data Collection, Analysis, and Modeling of the the Cas12h1^WT^-crRNA-dsDNA interference complex.** **a,** A representative raw cryo-EM micrograph of the interference complex. **b**, Representative 2D class averages. **c,** Classification strategy and refined maps of the interference complex. About 3.5 million particles were auto-picked, and after 2D classification, a total of 534,801 particles for further 3D classification. The homogeneous refinement with 153,309 particles from best class was performed, resulting in a map at 3.00 Å resolution. **d,** Local resolution map for the reconstruction in (**c**). **e** Euler plot for interference complex. **f,** Plot of the global half-map FSC (solid red line). FSC plot for the reconstruction suggests an average resolution of 3.00 Å.

Supplementary Figure 6.


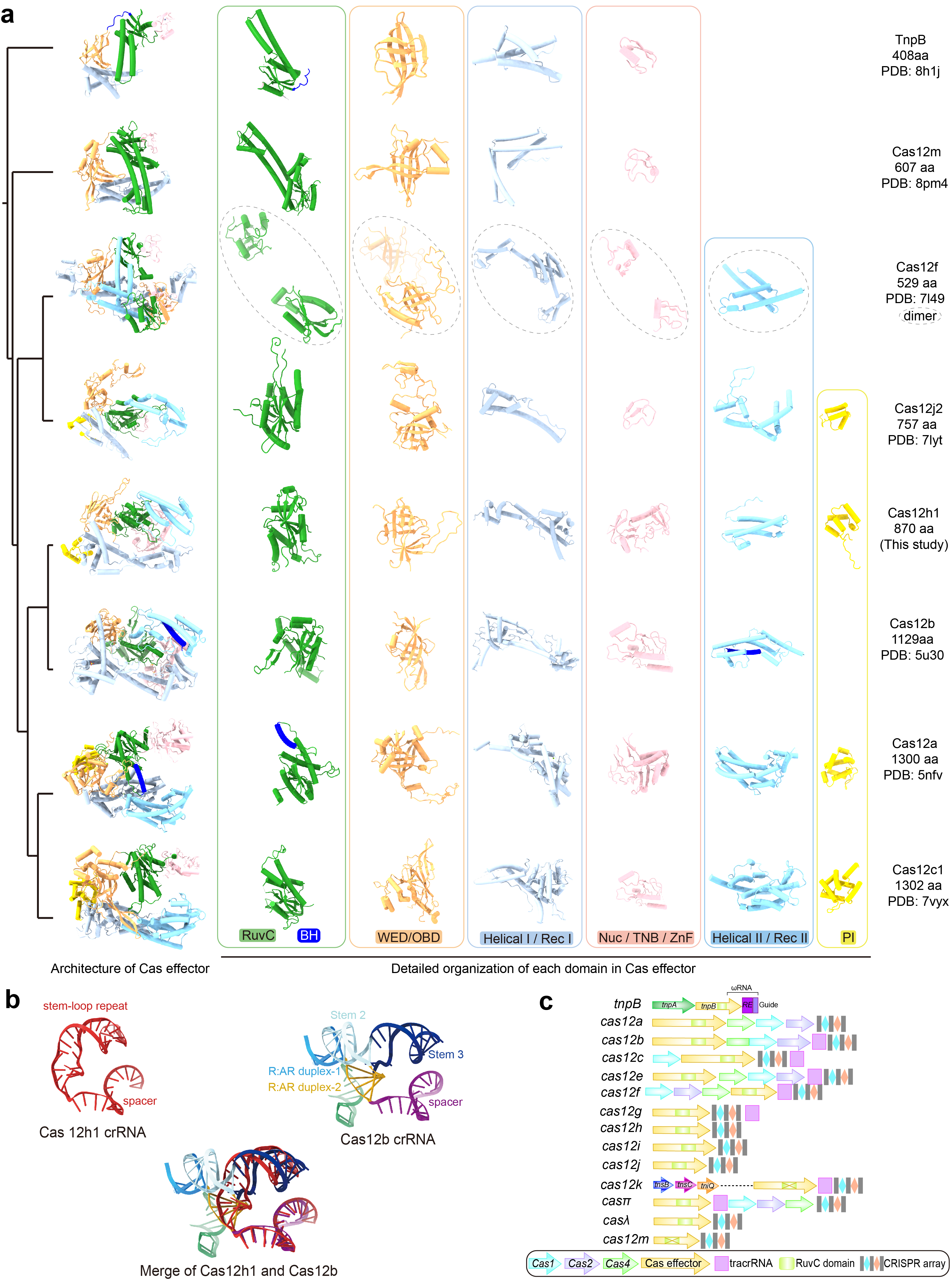


**Supplementary Figure 6. The portrait of type V Cas effectors. a,** Comparison of the domain organizations of type V Cas effectors with the phylogenetic tree shown on the left. The type V Cas effectors (each line for one effector) are sorted based on their evolutionary periods. The Cas proteins are in their interference state (from their ternary complexes) with the crRNA and target DNA hidden. The detailed organizations of the domains are shown on the right. Structures are shown as colored cartoons. Domains are color coded according to the legend on the bottom. **b,** Structure comparison of crRNA/gRNA of Cas12h1 and Cas12b. **c,** The representative CRISPR-*cas* loci of type V Cas effectors and TnpB.

Supplementary Figure 7.


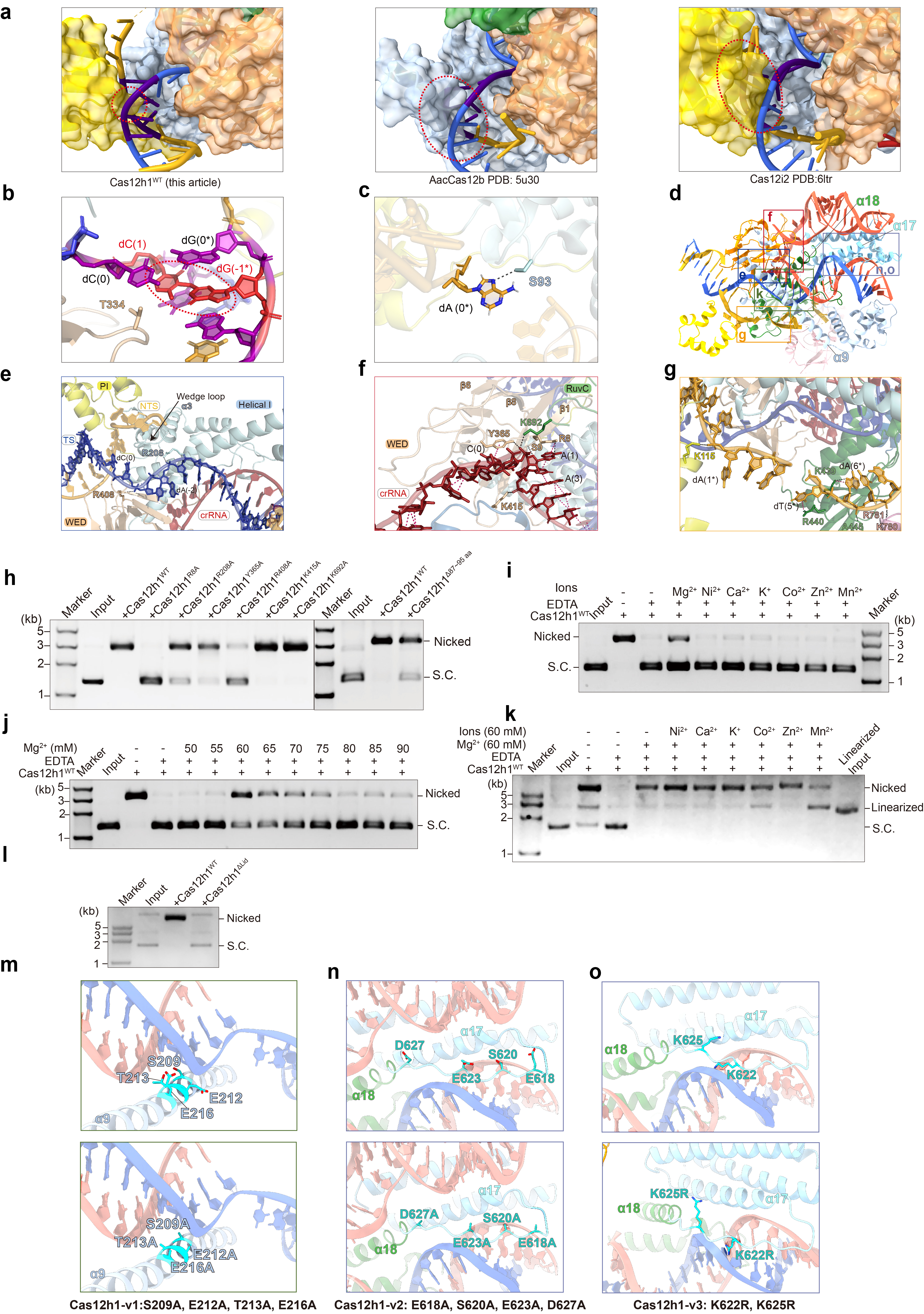


**Supplementary Figure 7. The architecture of PAM-interacting cleft and R-loop structure in interference state. a,** Comparison of structures of Cas12h1, Cas12b, and Cas12i2 in PAM region. A loop (Cas12b and Cas12i2) inserts into the minor groove of the PAM region. **b,** The modeled dG(-1*):dC(1) base pair would generate a steric clash between bases. No hydrogen bond retains between T334 and modelled nucleotides. **c,** A hydrogen bond between S93 and mutated dA(0*). **d,** The relative positions of (**e-g, k-m**) in the Cas12h1^WT^-crRNA-dsDNA interference complex. **e**, Detailed interactions of unwinding target duplex and Cas12h1 at the PAM-proximal region of the Cas12h1 interference complex. The wedge loop (residue 87~95aa) facilitates the unwinding of the target duplex. Hydrogen bonds between Cas12h1 and the unwound DNA are shown as black dashed lines. **f**, Detailed interactions of crRNA at the PAM-proximal region and Cas12h1. Hydrogen bonds between Cas12h1 and crRNA are shown as black dashed lines and pi-stacking interactions are shown as pink dashed lines. **g**, Detailed interactions between NTS DNA and Cas12h1 at PAM-proximal site. **h**, Supercoiled plasmid cleavage assay by wildtype Cas12h1 and its mutants. **i-k**, Investigation of the impact of metal ions on the cleavage ability of Cas12h1. The metal ions were removed by incubating Cas12h1^WT^-crRNA complex with EDTA, then the it was restored as indicated in the presence of EDTA. **i,** Supercoiled plasmid cleavage assay by wildtype Cas12h1 in the presence of EDTA and different metal ions. Each metal ion was added at a final concentration of 60 mM. **j,** Supercoiled plasmid cleavage assay by wildtype Cas12h1 investigating the impact of Mg^2+^ on cleavage ability. **k**, Supercoiled plasmid cleavage assay by wildtype Cas12h1 investigating the impact of Mg^2+^ in combination with other metal ions on cleavage ability. The reaction was terminated 2 hours after the addition of metal ions. **l**, Supercoiled plasmid cleavage assay by wildtype Cas12h1 and its mutant. **m-o**. The structures of Cas12h1 (upper panel) and its predicted structure of Cas12h1 variants (lower panel). **m**, Cas12h1-v1, **n**, Cas12h1-v2, **o**, Cas12h1-v3.

Supplementary Figure 8.


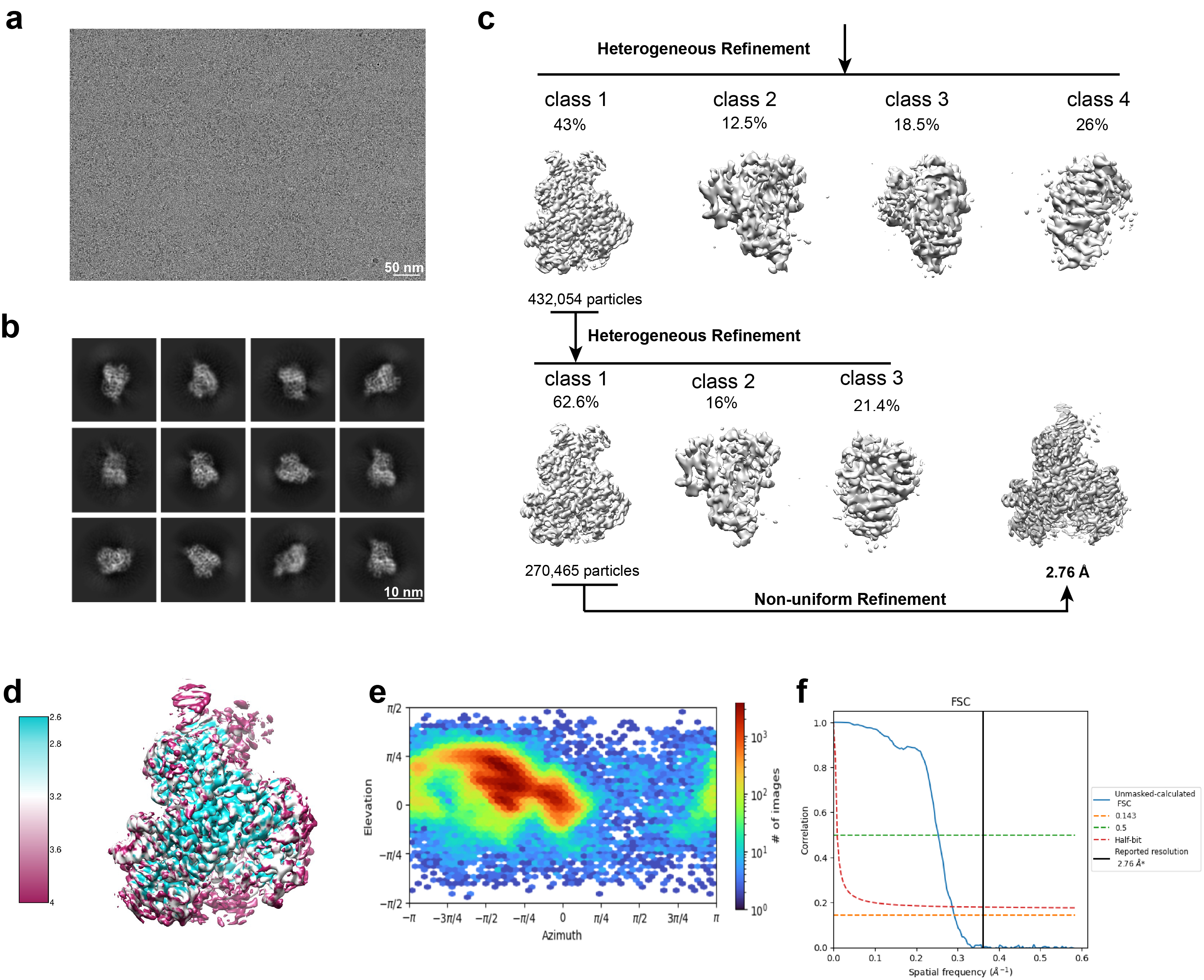


**Supplementary Figure 8. Cryo-EM Data Collection, Analysis, and Modeling of the Cas12h1^D465A^-crRNA-dsDNA R-loop formation complex.** **a,** A representative raw cryo-EM micrograph of the R-loop formation complex. **b**, Representative 2D class averages. **c,** Classification strategy and refined maps of the R-loop formation complex. About 3 million particles were auto-picked, and after 2D classification, a total of 432,054 particles for further 3D classification. The homogeneous refinement with 270,465 particles from best class was performed, resulting in a map at 2.76 Å resolution. **d,** Local resolution map for the reconstruction in (**c**). **e** Euler plot for Cas12h1^D465A^-crRNA-dsDNA R-loop formation complex. **f,** Plot of the global half-map FSC (solid red line). FSC plot for the reconstruction suggests an average resolution of 2.76 Å.

Supplementary Figure 9.


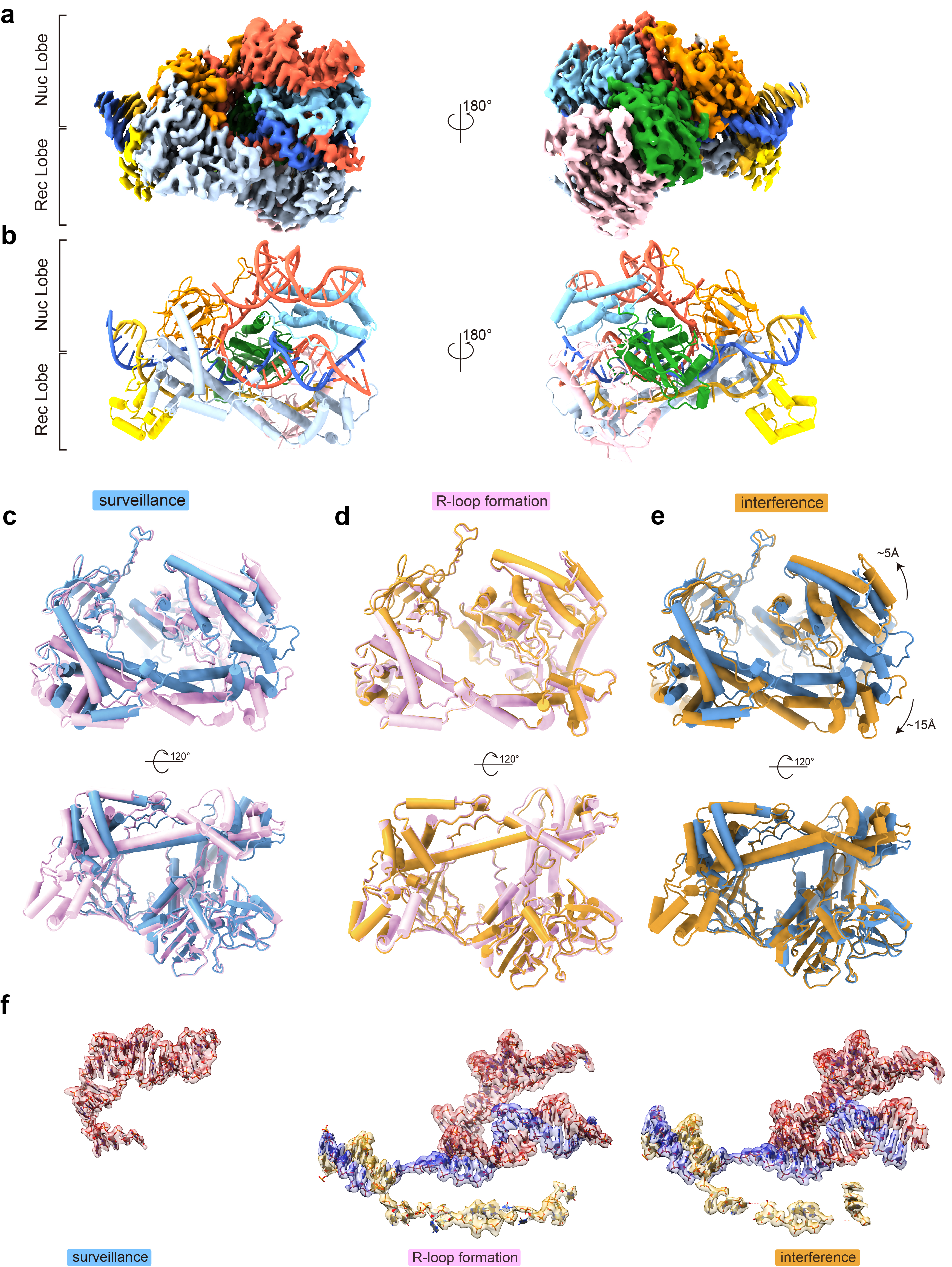


**Supplementary Figure 9. Comparison of the structures of Cas12h1 surveillance, R-loop formation, and interference state. a,** Cryo-EM map of the Cas12h1^D465A^-crRNA-dsDNA interference complex in one view (up) and the 180° rotated view (down). Domains of Cas12h1 are colored as in Figure **3a**. **b**, Atomic models of the Cas12h1^D465A^-crRNA-dsDNA interference complex in two views in cartoon. **c,** Superimposition of the protein structures of surveillance complex (blue) and R-loop formation complex (pink) and its 120° rotated view. **d**, Superimposition of the protein structures of R-loop formation complex (pink) and interference complex (orange) and its 120° rotated view. **e**, Superimposition of the protein structures of surveillance structure (blue) and R-loop formation complex (pink) and its 120° rotated view. **f,** Electron density maps of nucleotides in the Cas12h1 in surveillance, R-loop formation, and interference state, with atomic models fitted in.

Supplementary Figure 10.


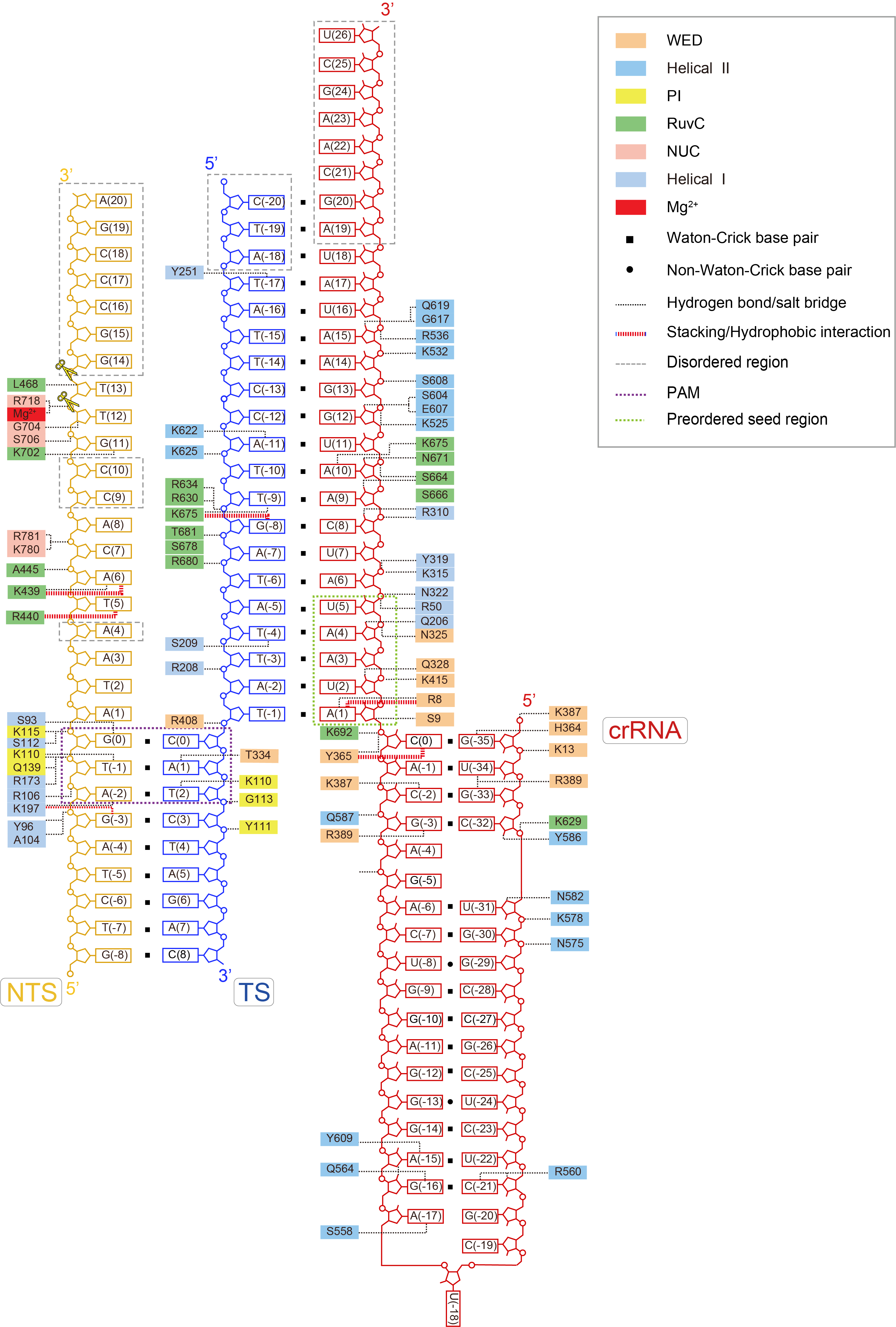


**Supplementary Figure 10. The interaction between Cas12h1 and nucleic acids.** Schematic of the R-loop recognition in the Cas12h1^WT^-crRNA-dsDNA interference structure. Domains and residues are colored according to Figure 2. Hydrogen bonds and salt bridges are shown as black dashed lines. Stackings and hydrophobic interactions are shown as red dashed lines. Disordered regions are encircled by gray dashed lines, PAM region is encircled by purple dashed lines, and crRNA seed region is encircled by green dashed lines.

**Supplementary Figure 11**

**
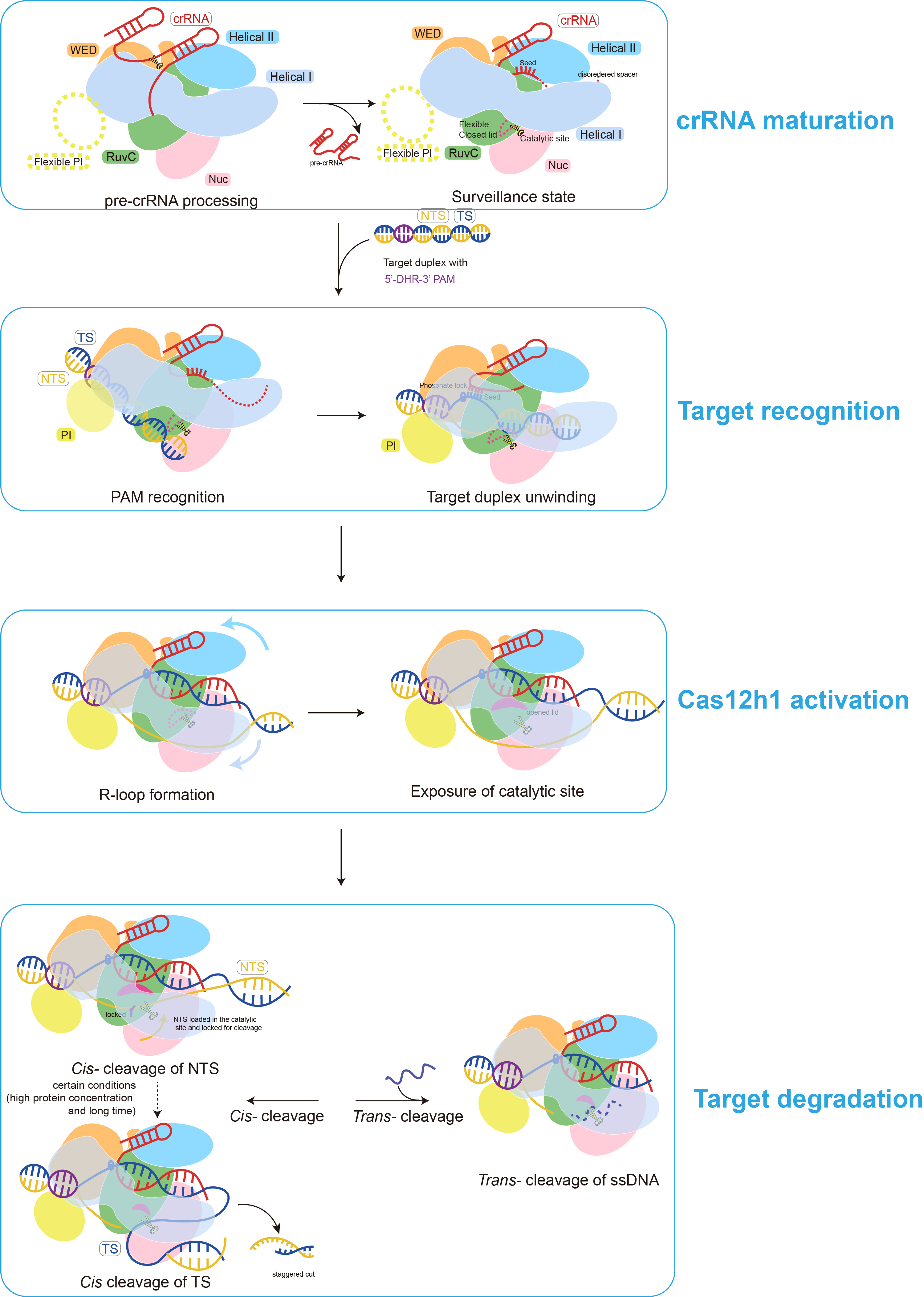
**

**Supplementary Figure 11. A model for the catalytic mechanism of Cas12h1.** Cas12h1 processes pre-crRNA into mature crRNA and forms a surveillance complex with mature crRNA, where the PI domain is flexible. The spacer region of the crRNA is also flexible except a 5 nt pre-ordered seed region. When matched with a 5’-DHR-3’ PAM, the PI domain is stabilized and together with the WED and Helical I domain, forms a PAM-interacting cleft. Beyond PAM recognition, the target duplex downstream of PAM sequence unwinds, and the residue R408 acts as a phosphate lock that stabilizes the unwinding target duplex. The TS DNA is guided towards the seed region of crRNA and further forms a 20bp heteroduplex with crRNA in the premise of correctly matching with the seed region. Formation of the heteroduplex triggers the Helical I and Helical II domain conformational rearrangements, and further lifts up and stabilizes the previously flexible lid loop in an “open” conformation for the activation of DNase activity. The unwound NTS DNA is guided through the groove formed by the RuvC and Nuc domain to the catalytic site for first-strand cleavage, and the residue K702 acts as a lock that stabilizes the NTS to facilitate target degradation. After the cleavage of the NTS, the TS disassociated with the crRNA from the 18^th^ nucleotide and loaded to catalytic site for second strand cleavage when the protein concentration is high and treated for a long time. If available, the active Cas12h1 also indiscriminately degrades ssDNA in *trans*.

**Supplementary Figure 12**

**
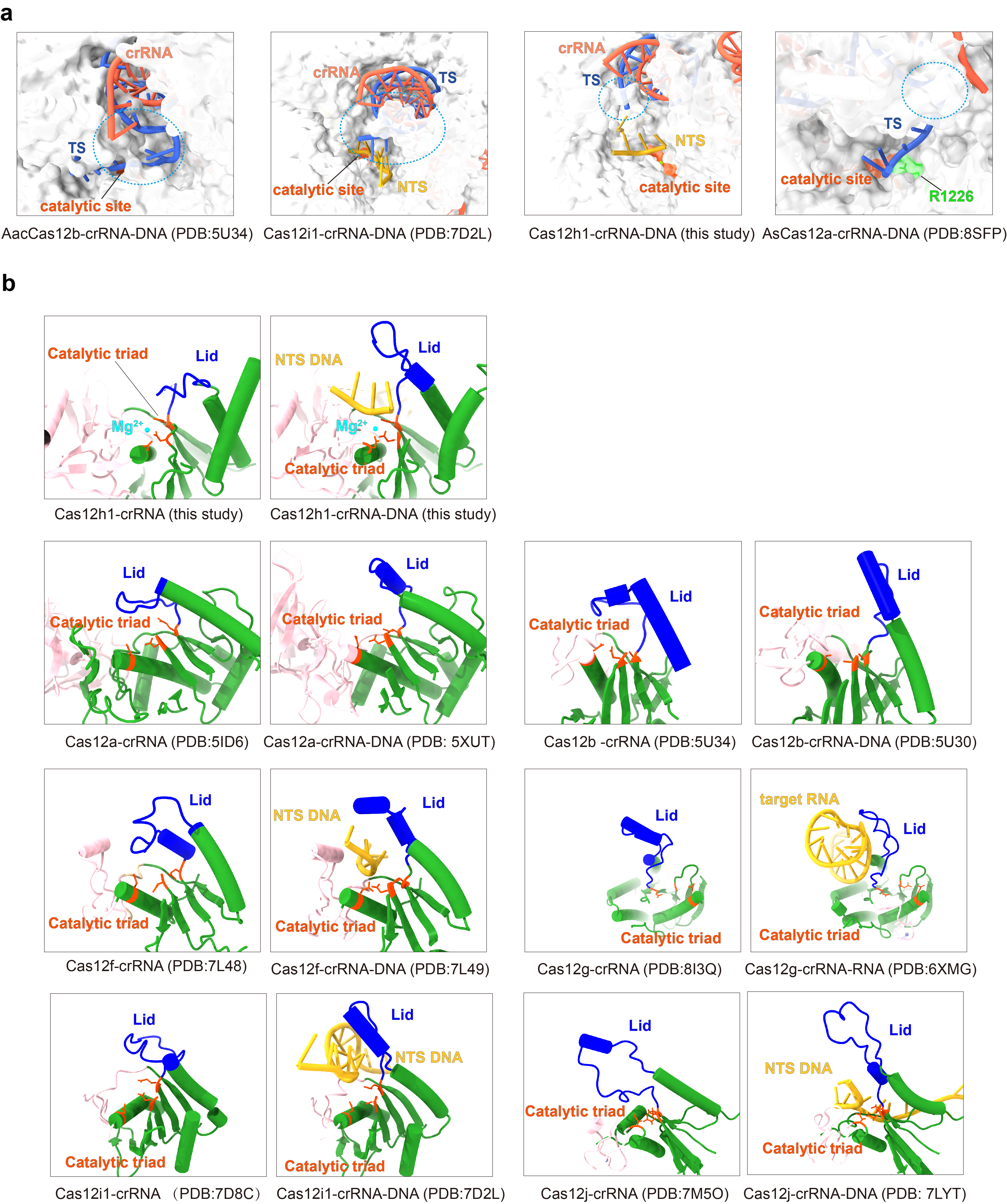
**

**Supplementary Figure 12. Comparison of Cas12h1 and other type V Cas effectors. a,** Protein-nucleotides interaction at PAM-distal heteroduplex. The twisted TS is highlighted in blue dashed circle. **b,** The lid motif (colored in blue) before and after DNA substrate recognition in Cas12h1, Cas12a, Cas12b, Cas12f, Cas12g, Cas12i1 and Cas12j2 are shown in parallel. Left panels are the structures without substrate, while right panels are structures with substrate. The D-E-D catalytic triad is highlighted in orange red. The lid motif undergoes a “loop to helix” (Cas12a, Cas12b, Cas12f, Cas12i) or a “helix to loop” (Cas12g, Cas12j2) transition during activation, and rotate up to accommodate the substrate. The lid in Cas12h1 undergoes a “flexible to stable” transition and rotate up for activation.

Supplementary Table 1. Statistics of cryo-EM data of Cas12h1 complex and structure refinement.

|  | **Cas12h1-crRNA binary complex** | **Cas12h1^WT^-crRNA-dsDNA ternary complex** | **Cas12h1^D465A^-crRNA-dsDNA ternary complex** |
| --- | --- | --- | --- |
| **Data collection** |  |  |  |
| EM | Titan Krios 300 kV,  K3 Gatan Summit | Titan Krios 300 kV,  K3 Gatan Summit | Titan Krios 300 kV,  K3 Gatan Summit |
| Magnification | 105,000 | 105,000 | 105,000 |
| Pixel size (Å) | 0.855 | 0.855 | 0.855 |
| Electron exposure(e−/Å^2^) | 50 | 50 | 50 |
| Defocus range (μm) | -1.2 to -1.8 | -1.2 to -1.8 | -1.2 to -1.8 |
| Initial particle images (no.) | 679,696 | 534,801 | 432,054 |
| Final particle images (no.) | 86,736 | 153,309 | 270,465 |
| Final resolution (Å) | 3.00 | 3.00 | 2.76 |
| **Model composition** |  |  |  |
| Protein residues | 760 | 851 | 853 |
| Nucleic acids | 41 | 99 | 107 |
| **B factors (Å^2^)** |  |  |  |
| Protein residues | 28.09/128.59/74.33 | 30.43/114.73/57.95 | 30.00/185.37/85.95 |
| Nucleic acids | 43.39/119.62/86.40 | 20.00/85.91/35.63 | 20.00/162.88/96.59 |
| **R.m.s.deviations** |  |  |  |
| Bond lengths (Å) | 0.005(7) | 0.012(74) | 0.004 (1) |
| Bonds angles (°) | 0.692 (19) | 1.172(50) | 0.698 (1) |
| **Validation** |  |  |  |
| MolProbity score | 1.69 | 1.83 | 1.71 |
| Clash score | 11.11 | 6.18 | 7.88 |
| **Ramachandran plot** |  |  |  |
| Favored (%) | 97.33 | 97.40 | 95.99 |
| Allowed (%) | 2.67 | 2.60 | 4.01 |
| Outliers region (%) | 0.00 | 0.00 | 0.00 |

Supplementary Table 2 Plasmid used in this study

| **Plasmids** | **Template Sequence** | **Back-bone** | **Cloning site** |
| --- | --- | --- | --- |
| Cas12h1^WT^  *E.coli* codon-optimized | ATGAAAGTGCACGAAATTCCGCGTAGCCAGCTGCTGAAAATTAAACAGTACGAAGGCAGCTTTGTTGAATGGTACCGCGACCTGCAGGAAGATCGCAAAAAGTTTGCCAGCCTGCTGTTTCGTTGGGCAGCATTCGGTTATGCAGCACGTGAAGACGACGGTGCAACCTATATTTCACCGAGTCAGGCGCTGCTGGAACGTCGCCTGCTGCTGGGTGACGCCGAAGATGTTGCTATTAAATTTCTGGATGTTCTGTTTAAAGGCGGTGCACCGAGCAGCAGTTGCTATAGCCTGTTTTATGAGGATTTTGCTCTGCGTGACAAAGCCAAATATAGCGGTGCAAAACGTGAATTTATTGAGGGTCTGGCAACCATGCCGCTGGATAAAATCATTGAGCGTATTCGTCAGGATGAACAGCTGAGCAAAATTCCGGCAGAAGAATGGCTGATTCTGGGGGCAGAATATAGCCCGGAAGAAATTTGGGAACAGGTGGCACCGCGTATCGTGAACGTTGATCGCAGCCTGGGTAAACAGCTGCGCGAACGCCTGGGTATTAAATGCCGTCGTCCTCATGATGCAGGGTATTGTAAAATTCTGATGGAAGTTGTTGCACGTCAACTGCGTAGCCATAATGAAACATATCATGAATATCTGAACCAGACCCATGAGATGAAAACTAAAGTTGCAAACAATCTGACAAACGAATTCGACCTGGTGTGTGAGTTTGCAGAAGTCCTGGAAGAAAAAAATTATGGTCTGGGTTGGTATGTTCTGTGGCAGGGTGTTAAACAGGCACTGAAAGAACAGAAAAAACCGACCAAAATTCAGATTGCAGTTGATCAGCTGCGTCAGCCGAAATTTGCAGGTCTGCTGACCGCAAAATGGCGTGCACTGAAAGGTGCATACGATACCTGGAAACTGAAAAAACGTCTGGAAAAACGTAAAGCATTTCCGTATATGCCGAATTGGGATAATGATTATCAGATTCCGGTTGGTCTGACCGGTCTGGGTGTTTTTACCCTGGAAGTTAAACGTACCGAAGTTGTTGTTGATCTGAAAGAACATGGTAAACTGTTTTGTAGCCATAGCCATTATTTTGGTGATCTGACCGCAGAAAAACATCCGAGCCGTTATCATCTGAAATTTCGTCATAAACTGAAACTGCGTAAACGTGATAGCCGTGTTGAACCGACCATTGGTCCGTGGATTGAAGCAGCACTGCGTGAAATTACCATTCAGAAAAAACCGAATGGTGTTTTTTATCTGGGTCTGCCGTATGCACTGAGCCATGGTATTGATAATTTTCAGATTGCAAAACGTTTTTTTAGCGCAGCAAAACCGGATAAAGAAGTTATTAATGGTCTGCCGAGCGAAATGGTTGTTGGTGCAGCAGATCTGAATCTGAGCAATATTGTTGCACCGGTTAAAGCACGTATTGGTAAAGGTCTGGAAGGTCCGCTGCATGCACTGGATTATGGTTATGGTGAACTGATTGATGGTCCGAAAATTCTGACCCCGGATGGTCCGCGTTGTGGTGAACTGATTAGCCTGAAACGTGATATTGTTGAAATTAAAAGCGCAATTAAAGAATTTAAAGCATGTCAGCGTGAAGGTCTGACCATGAGCGAAGAAACCACCACCTGGCTGAGCGAAGTTGAAAGCCCGAGCGATAGCCCGCGTTGTATGATTCAGAGCCGTATTGCAGATACCAGCCGTCGTCTGAATAGCTTTAAATATCAGATGAATAAAGAAGGTTATCAGGATCTGGCAGAAGCACTGCGTCTGCTGGATGCAATGGATAGCTATAATAGCCTGCTGGAAAGCTATCAGCGTATGCATCTGAGCCCGGGTGAACAGAGCCCGAAAGAAGCAAAATTTGATACCAAACGTGCAAGCTTTCGTGATCTGCTGCGTCGTCGTGTTGCACATACCATTGTTGAATATTTTGATGATTGTGATATTGTTTTTTTTGAAGATCTGGATGGTCCGAGCGATAGCGATAGCCGTAATAATGCACTGGTTAAACTGCTGAGCCCGCGTACCCTGCTGCTGTATATTCGTCAGGCACTGGAAAAACGTGGTATTGGTATGGTTGAAGTTGCAAAAGATGGTACCAGCCAGAATAATCCGATTAGCGGTCATGTTGGTTGGCGTAATAAACAGAATAAAAGCGAAATTTATTTTTATGAAGATAAAGAACTGCTGGTTATGGATGCAGATGAAGTTGGTGCAATGAATATTCTGTGTCGTGGTCTGAATCATAGCGTTTGTCCGTATAGCTTTGTTACCAAAGCACCGGAAAAAAAAAATGATGAAAAAAAAGAAGGTGATTATGGTAAACGTGTTAAACGTTTTCTGAAAGATCGTTATGGTAGCAGCAATGTTCGTTTTCTGGTTGCAAGCATGGGTTTTGTTACCGTTACCACCAAACGTCCGAAAGATGCACTGGTTGGTAAACGTCTGTATTATCATGGTGGTGAACTGGTTACCCATGATCTGCATAATCGTATGAAAGATGAAATTAAATATCTGGTTGAAAAAGAAGTTCTGGCACGTCGTGTTAGCCTGAGCGATAGCACCATTAAAAGCTATAAAAGCTTTGCACATGTTTAA | pET-30b | *Nde*I- *Xho*I |

| **Plasmids** | **Template Sequence** | **Back-bone** | **Cloning site** | |
| --- | --- | --- | --- | --- |
| Cas12h1 CRISPR RNA | GGGGAATTGTGAGCGGATAACAATTCCCCATCTTAGTATATTAGTTAAGTATAAGAAGGAGATATACATATGGTGCTGGCCGCTCTCGCTAGAGGGAGGTCAGAGCACATAATATCAATGGAATATAGCAAGCTGTGCTGGCCGCTCTCGCTAGAGGGAGGTCAGAGCACATAATATCAATGGAATATAGCAAGCTGTGCTGGCCGCTCTCGCTAGAGGGAGGTCAGAGCACATAATATCAATGGAATATAGCAAGCTGTGCTGGCCGCTCTCGCTAGAGGGAGGTCAGAGCACATAATATCAATGGAATATAGCAAGCTGTGCTGGCCGCTCTCGCTAGAGGGAGGTCAGAGCACATAATATCAATGGAATATAGCAAGCTGTGCTGGCCGCTCTCGCTAGAGGGAGGTCAGAGCACATAATATCAATGGAATATAGCAAGCTGTGCTGGCCGCTCTCGCTAGAGGGAGGTCAGAGCACATAATATCAATGGAATATAGCAAGCTGTGCTGGCCGCTCTCGCTAGAGGGAGGTCAGAGCACCTCGAGTCTGGTAAAGAAACCGCTGCTGCGAAATTTGAACGCCAGCACATGGACTCGTCTACTAGCGCAGCTTAATTAACCTAGGCTGCTGCCACCGCTGAGCAATAA | pCDF-Duet-1 | *Nde*I- *Xho*I |  |
| Substrate for Cas12h1 | ATGATAATATCAATGGAATATAGCAAGCTCCCCCCC | pUC-19 | *Xba*I-*Bam*HI |  |
| Guide-s for Cas12h1 in EGFP activation | GTGCTGGCCGCTCTCGCTAGAGGGAGGTCAGAGCACGGTGAGTGAGTGTGTGCGTGTTTTTT | pLentiCRISPR-v2 | *Bsm*BI |  |
| Guide-as for Cas12h1 in EGFP activation | GTGCTGGCCGCTCTCGCTAGAGGGAGGTCAGAGCACCACGCACACACTCACTCACCTTTTTT | pLentiCRISPR-v2 | *Bsm*BI |  |
| Guide for Cas9 in EGFP activation | GGTGAGTGAGTGTGTGCGTG | pLentiCRISPR-v2 | *Bsm*BI |  |

Supplementary Table 3. Fluorescently labeled oligonucleotides in this study

| Oligo names | **Sequence (5′→3′)** |
| --- | --- |
| 3′-6FAM-TS | GGGGGAGCTTGCTATATTCCATTGATATTATCATCTAGAC-6FAM-3′ |
| 3′-6FAM-ladder-30nt | GCTATATTCCATTGATATTATCATCTAGAC-6FAM-3′ |
| 3′-6FAM-ladder-31nt | TGCTATATTCCATTGATATTATCATCTAGAC-6FAM-3′ |
| 3′-6FAM-ladder-32nt | TTGCTATATTCCATTGATATTATCATCTAGAC-6FAM-3′ |
| 3′-6FAM-ladder-33nt | CTTGCTATATTCCATTGATATTATCATCTAGAC-6FAM-3′ |
| 3′-6FAM-ladder-34nt | GCTTGCTATATTCCATTGATATTATCATCTAGAC-6FAM-3′ |
| 5′-6FAM-TS | 5′-6FAM-GTCTAGATGATAATATCAATGGAATATAGCAAGCTCCCCC |
| 5′-6FAM-ladder-20nt | 5′-6FAM-GTCTAGATGATAATATCAAT |
| 5′-6FAM-ladder-21nt | 5′-6FAM-GTCTAGATGATAATATCAATG |
| 5′-6FAM-ladder-22nt | 5′-6FAM-GTCTAGATGATAATATCAATGG |
| 5′-6FAM-ladder-23nt | 5′-6FAM-GTCTAGATGATAATATCAATGGA |
| 5′-6FAM-ladder-24nt | 5′-6FAM-GTCTAGATGATAATATCAATGGAA |
| 5′-6FAM-non-specific-ssDNA-50nt | 5′-6FAM-ATATATATATATATATATATAATATATATATATATATATATAATATATAT |
| FQ reporter | 5'6-FAM -TTATTATTAT-3'BHQ |

Data S1. (separate file)

Original Gel pictures in the figures.


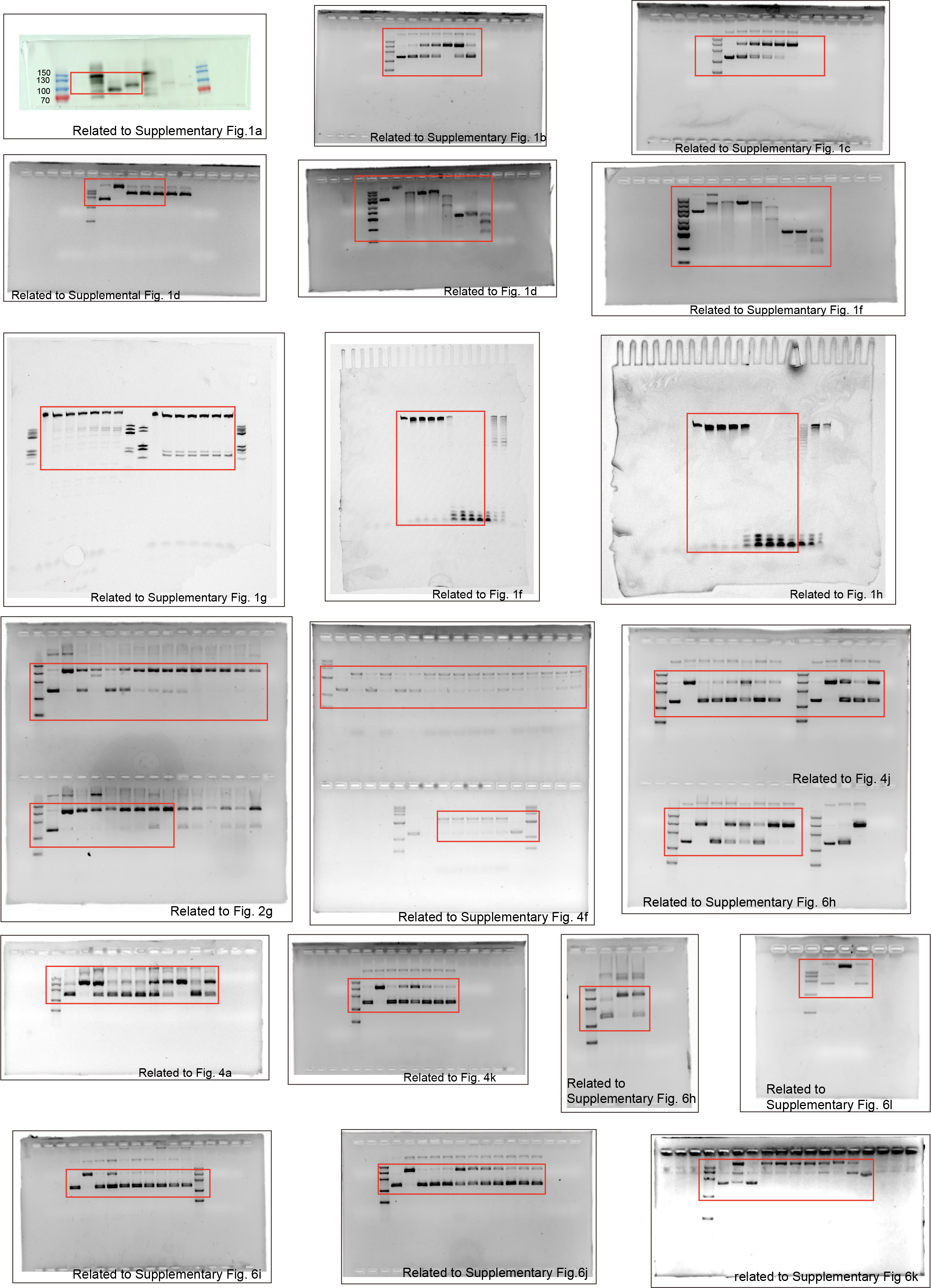

Supplement: Supplementary file 1 — Supplemntary Files [file 41392_2025_2147_MOESM1_ESM.docx]
